# Factors influencing the implementation of cardiovascular risk scoring in primary care: a mixed-method systematic review

**DOI:** 10.1186/s13012-020-01022-x

**Published:** 2020-07-20

**Authors:** Tonny B. Muthee, Derick Kimathi, Georgia C. Richards, Anthony Etyang, David Nunan, Veronika Williams, Carl Heneghan

**Affiliations:** 1 Centre for Evidence-Based Medicine, Nuffield Department of Primary Care Health Sciences, University of Oxford, Woodstock Road, Oxford, OX2 6GG UK; 2 KEMRI-Wellcome Trust Research Programme, Nuffield Department of Medicine, University of Oxford, Oxford, UK; 3 Epidemiology and Demography Department, KEMRI-Wellcome Trust Research Programme, Kilifi, Kenya; 4 Faculty of Education and Professional Studies, School of Nursing, Nipissing University, North Bay, Canada

**Keywords:** Cardiovascular, Risk, Assessment, Scoring, Facilitators, Barriers, Implementation, Primary care, Mixed methods

## Abstract

**Background:**

Cardiovascular disease (CVD) such as ischemic heart disease and stroke is the leading causes of death and disability globally with a growing burden in low and middle-income countries. A credible way of managing the incidence and prevalence of cardiovascular diseases is by reducing risk factors. This understanding has led to the development and recommendation for the clinical use of cardiovascular risk stratification tools. These tools enhance clinical decision-making. However, there is a lag in the implementation of these tools in most countries. This systematic review seeks to synthesise the current knowledge of the factors influencing the implementation of cardiovascular risk scoring in primary care settings.

**Methods:**

We searched bibliographic databases and grey literature for studies of any design relating to the topic. Titles, abstracts and full texts were independently assessed for eligibility by two reviewers. This was followed by quality assessment and data extraction. We analysed data using an integrated and best fit framework synthesis approach to identify these factors. Quantitative and qualitative forms of data were combined into a single mixed-methods synthesis. The Consolidated Framework for Implementation Research was used as the guiding tool and template for this analysis.

**Results:**

Twenty-five studies (cross-sectional *n* = 12, qualitative *n* = 9 and mixed-methods *n* = 4) were included in this review. Twenty (80%) of these were conducted in high-income countries. Only four studies (16%) included patients as participants. This review reports on a total of eleven cardiovascular risk stratification tools. The factors influencing the implementation of cardiovascular risk scoring are related to clinical setting and healthcare system (resources, priorities, practice culture and organisation), users (attributes and interactions between users) and the specific cardiovascular risk tool (characteristics, perceived role and effectiveness).

**Conclusions:**

While these findings bolster the understanding of implementation complexity, there exists limited research in the context of low and middle-income countries. Notwithstanding the need to direct resources in bridging this gap, it is also crucial that these efforts are in concert with providing high-quality evidence on the clinical effectiveness of using cardiovascular risk scoring to improve cardiovascular disease outcomes of mortality and morbidity.

**Trial registration:**

PROSPERO registration number: CRD42018092679.

Contributions to the literature

- Factors influencing the implementation of cardiovascular risk scoring in primary care settings buttress the general understanding that implementation processes are complex, intricately related and dependent on context.
- More research on the use of cardiovascular risk prediction tools is needed in low and middle-income countries in the form of effectiveness-implementation hybrid designs which combine the elements of clinical effectiveness and implementation research to enhance public health impact.
- The use of an integrated best fit framework synthesis allowed us to position our findings in a widely used implementation science framework: CFIR.

## Background

Cardiovascular disease (CVD) such as ischemic heart disease and stroke is the leading causes of death and disability globally, with a growing burden, particularly in low and middle-income countries [1–5]. While unmodifiable risk factors (age, sex, race, genetics and familial history) contribute to CVD burden, most contributing risk factors are modifiable such as abnormal cholesterol levels, elevated blood pressure, smoking, unhealthy diet, moderate/excessive alcohol intake, sedentary lifestyle and psychosocial stress. It is estimated that these risk factors contribute up to 90% of the attributable risk for ischemic heart disease and stroke in the global population [6].

Cardiovascular risk scoring allows health care professionals to make clinical decisions by quantitatively predicting the absolute risk of a cardiovascular event occurring during a defined period by using risk scoring tools. These tools are widely accepted as the mainstay in cardiovascular disease prevention guidelines [6–9]. Systematic reviews have shown that providing cardiovascular risk scores has an impact on patients at risk of CVD by increasing the prescribing of lipid-lowering and blood-pressure-lowering medication in higher-risk patients, and reducing cardiovascular risk estimates in low-risk patients [10–12].

The reduction of risk factors (number or magnitude) in any individual, albeit on a lesser degree, is beneficial. Geoffrey Rose’s prevention theory explains that small reduction in the magnitude of a risk factor at the population level (low-to-moderate risk), resulting in a leftward shift in the overall population risk distribution, results in fewer cases of disease than reducing the risk factor by even a large magnitude in those at elevated risk [13]. Besides, cardiovascular risk tools have also been shown to escalate prevention efforts by matching these efforts to a person’s absolute risk of developing a cardiovascular event [1]. This, therefore, helps to direct patients at elevated risk of developing cardiovascular disease into treatment as they would benefit more compared to those at low risk; to whom the harms of medication might outweigh the benefits [6, 8, 14, 15]. The subsequent use of these tools suggests an overall reduction of a patient’s absolute cardiovascular risk by reducing the magnitude of their pre-treatment risk [6]. There is no high-quality evidence yet, as to whether the use of these tools influences cardiovascular outcomes on mortality; however, their usefulness in the reduction of cardiovascular risk factors is well reported.

Notwithstanding that CVD risk tools are recommended in most clinical guidelines as the mainstay for cardiovascular disease prevention worldwide, a global survey conducted by the WHO in 2015 reported a lag in the implementation of these tools in most countries [16]. This is despite progress in infrastructure, governance, financing, policies, action plans, strategies, surveillance and non-communicable disease management. Only 21% of the countries reported having more than 50% of primary health care facilities offering cardiovascular risk stratification for high-risk patients. In 26% of the countries, the use of cardiovascular risk stratification for these patients was in less than 25% of the primary care facilities. There was significantly more use of this intervention in high-income countries (> 35%) compared to low and middle-income countries. In low and middle-income countries, 42% of these countries offered no risk stratification in any of their primary facilities.

There is not enough implementation research to explain these disparities, especially in low and middle-income countries. This review seeks to synthesise the current knowledge of the factors influencing the implementation of cardiovascular risk scoring as a cardiovascular disease prevention strategy in primary care settings. This understanding could guide implementation policy and practice and provide insight as to how cardiovascular risk assessment tools can be best utilised in various settings.

## Methods

This review has been reported following the Preferred Reporting Items for Systematic Reviews (PRISMA) guidelines for 2015 [17] [see Table S1 in Additional File 1].

### Search strategy

A systematic search was carried out to identify all the relevant studies that reported on facilitators and barriers to cardiovascular risk scoring in primary care. The full search strategy is available in Additional File 2***.*** This strategy was adapted and ran in the following databases: EMBASE, MEDLINE, the Cochrane Library (CENTRAL), CINAHL, PsycINFO, Global Health, Web of Science and in Grey literature. The search was not limited by date or language. Also, we searched references of all included studies alongside forward and backward citation searching of key articles already known to us.

### Selection criteria

For inclusion, studies had to report on factors relating to increased, decreased or no use of total cardiovascular risk assessment tools. This included aspects that influenced the usability and adoption of these tools, opportunity costs and resources associated with their use or/and adoption. Only primary studies, i.e. qualitative, quantitative and mixed-method, were included. Studies of mixed populations in primary and secondary care were only included when there was access to data exclusively from primary care populations. Where it was not clear, clarification was sought from the authors. Only studies that assessed the systematic provision of an absolute cardiovascular risk assessment, by the integration of three or more risk factors to a patient by a physician or a non-physician as a strategy for primary prevention of cardiovascular disease, were included.

Studies were excluded if they involved participants less than 18 years old with pre-existing cardiovascular disease. Studies that assessed cardiovascular risk using two or fewer risk factors were excluded. Those that assessed self-administration of total cardiovascular risk assessment score via online calculators or charts were also excluded.

At first instance, TBM screened all the studies retrieved for title and abstract screening while DK and GCR independently screened half of these studies each. The full-text screening was done by two reviewers (TM and GCR) where disagreements were settled by consensus or by consulting the third reviewer (DK). A record of the excluded studies after full-text screening and the reasons for exclusion is available in Table S3 in Additional File 3. The decision process leading to the included studies was documented on a PRISMA flow chart [see Figure S1 in Additional File 4]*.* This selection process was managed using Covidence [18].

### Assessing the quality of the studies

The methodological quality of mixed-method studies was assessed using the Mixed Methods Appraisal Tool (MMAT), and for qualitative studies, the critical appraisal checklist developed by the National Institute for Health and Care Excellence (NICE) for qualitative studies was used [19, 20]. For the quantitative studies, all of which were cross-sectional studies, the AXIS Appraisal Tool was used [21]. Two reviewers (TM and GCR) independently appraised all the studies, and disagreements were resolved by consensus.

### Data extraction strategy

The characteristics of the included studies, i.e. the country’s Gross National Income classification, study design, demographics of the participants, type of cardiovascular assessment tool and the study outcomes were extracted independently and in duplicate by TM and GR using Microsoft Excel 2016. Discrepancies were resolved by consensus.

### Data synthesis and presentation

**Fig. 1:**
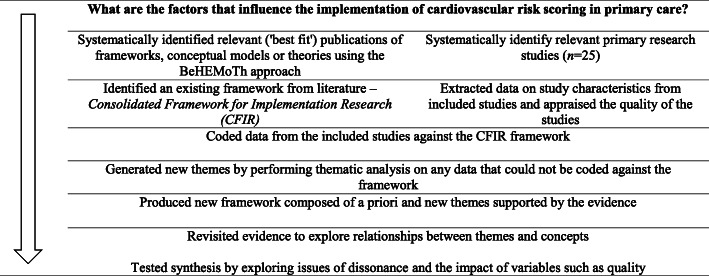
Steps to using a ‘Best fit’ framework synthesis

Synthesis took a ‘Best fit’ framework synthesis approach and utilised an integrated mixed-methods methodology (Fig. 1) [22, 23]. The ‘Best fit’ framework synthesis, an approach based on the framework analysis technique, was then used to identify the facilitators and barriers. Figure 1 explains the seven steps of the approach that allowed for an inductive and deductive approach to data synthesis. An integrated mixed-methods methodology involved ‘combining’ qualitative and quantitative data by having quantitative data contribute to the thematic typology.

After identifying the relevant primary studies for the review, a priori (‘best fit’) framework was similarly identified from existing frameworks in the literature using the BeHEMoTh (Behaviour of interest, Health context, Exclusions and Models or Theories) strategy. This strategy focused on implementation science theories and frameworks. Consequently, the Consolidated Framework for Implementation (CFIR) was identified as the ‘best fit’ framework (Table 1). The CFIR is a multilevel determinant framework that draws on psychological and organisational theories [24]. It subsumes its multiple constructs into five domains, as shown in Table 1. Data extracted from the primary studies were sorted and indexed onto this framework.

**Table 1:** An outline of the Consolidated Framework of Implementation Research (CFIR)

	CFIR domains	CFIR constructs
Implentation	Intervention	Intervention source, evidence strength and quality, relative advantage, adaptability, trialability, complexity, design quality and packaging and cost
	Outer setting	Patients’ needs and resources, cosmopolitanism, peer pressure and external policy and incentives
	Inner setting	Structural characteristics, networks and communications, culture, implementation climate and readiness for implementation
	Individuals	Knowledge and belief about the intervention, self-efficacy, individual stage of change, individual identification with the organisation and other personal attributes.
	Process	Planning, engaging, executing, reflecting and evaluating

For quantitative and mixed methods studies, data on the facilitators and barriers were extracted on an Excel spreadsheet. This data was then converted and thematically coded under the CFIR constructs outlined above. This conversion process is adapted from the Joanna Briggs Institute methodology for aggregative mixed-method synthesis [25]. In this conversion, quantitative data lends itself to the derivation of defined themes which can then be coded. All data, both the converted and those from qualitative studies, were then coded using NVivo 12 onto the 39 constructs of the CFIR [see Table S4 in Additional File 5]*.*

### Additional analyses

The variations between the facilitators and barriers across countries’ Gross National Income Indices and the users of cardiovascular risk tools were identified, categorised and explored.

In addition, the confidence/certainty of the review findings were assessed to strengthen the rigour of our review. Each review finding was assessed using the Confidence in the Evidence from Reviews of Qualitative Research (CERQual) approach [26]. CERQual is an innovative approach that provides a systematic and transparent framework in assessing confidence in individual qualitative review findings. This assessment is based on four components: methodological limitations, coherence, adequacy of data and relevance [27–31]. In this review, the ‘confidence’ of a finding is the assessment of its ability to represent the phenomenon of interest reasonably, such that this finding does not differ substantially from the phenomenon of interest [26]. These four components were assessed as having minor, moderate or serious concerns. An overall assessment on the confidence in the evidence for each finding, based on these four components, was assessed as high, moderate or low [26].

Notably, this assessment was only carried out for individual findings from qualitative studies. This is because the CERQual approach is designed to assess the confidence of the individual conclusions in a qualitative review synthesis as opposed to a mixed-method review synthesis. However, there is an acknowledgement of concern in that, in assessing the qualitative elements of this mixed-methods review separately, there is a risk of undervaluing the contribution of the review findings based on integrated data. To alleviate this, the results of this assessment are adjunct to the main conclusions of the review.

TM conducted these additional analyses in consultation with VW.

## Results

### Study characteristics

After removing duplicate studies, 3010 studies were screened for title and abstract and 52 studies for full text. Twenty-five studies were included in this review—twelve quantitative, nine qualitative and four mixed methods. A summary of the study characteristics is available in Table S2 in Additional File 3. Most (*n* = 20) of the included studies were conducted in high-income countries, with only five conducted in lower middle-income countries. There were no studies from low-income countries. All the studies reported on health care professionals’ perspectives on cardiovascular risk scoring but only five reported on patients’ perspectives. Most of the studies (*n* = 23) involved physicians with only four of the studies involving non-physicians, i.e. practice nurses, health promoters, pharmacists, practice managers, administrators and policymakers.

Eleven cardiovascular risk-scoring tools were used in the studies included (Table 2). Five included studies did not mention the specific cardiovascular risk scoring tool used in their evaluation but reported using an absolute cardiovascular risk assessment strategy in their methods.

**Table 2:** Cardiovascular risk scoring tools reported in the review

Tool (Acronym and definition)		Studies	Countries in which these tools were used
ESC SCORE risk charts	The European Society of Cardiology-Systematic COronary Risk Evaluation	[32–38]	Switzerland, Brazil, the USA, Greece, Chile, Venezuela, Portugal, The Netherlands, Central America (Costa Rica, Panama, El Salvador and Guatemala), Austria, Belgium, France, Germany, Ireland, Norway, Russia, Spain, Sweden, Switzerland, Turkey and the UK.
Framingham Risk Score		[32, 34–42]	Switzerland, Germany, Turkey, Brazil, the USA, Greece, Chile, Venezuela, Portugal, The Netherlands, Central America (Costa Rica, Panama, El Salvador and Guatemala), Austria, Belgium, Egypt, France, Norway, Russia, Spain, Sweden and the UK_._
Framingham Risk Score—Modified		[32, 35, 37]	Turkey, Austria, Belgium, France, Germany, Greece, Norway, Russia, Spain, Sweden, Switzerland and the UK
PRECARD	A program for the Copenhagen Risk Score	[43]	Denmark
Cardiovascular Risk PROCAM Score	Cardiovascular Risk (Prospective Cardiovascular Munster) Score	[34, 38]	Switzerland, Brazil, The USA, Greece, Chile, Venezuela, Portugal, The Netherlands, Central America (Costa Rica, Panama, El Salvador and Guatemala)
AGLA Risk Score (ARS)	A PROCAM–derived Swiss risk score	[34]	Switzerland
New-Zealand Risk Score		[34, 44–48]	Australia, Switzerland and New Zealand
Framingham - REGICOR	Registre Gironi del cor—A Spanish adaptation of the Framingham	[39]	Spain
JBS Risk Calculator	Joint British Societies for the prevention of cardiovascular disease Risk Calculator	{39, 41, 45, 47, 50]	Australia, The UK, Brazil, The USA, Greece, Chile, Venezuela, Portugal, Germany, The Netherlands, Central America (Costa Rica, Panama, El Salvador and Guatemala) and Egypt
WHO/ISH cardiovascular risk prediction charts	The World Health Organisation and the International Society of Hypertension	[40, 49, 50]	Argentina, Egypt, and Jordan
QRisk		[40, 41]	The UK and Egypt

### Quality assessment of included studies

The response rates of all but one of the quantitative studies, a study by Tawfik et al. (2015), were inadequate—only two studies had a response rate above 50% [38, 39]. No information about non-responders was described in any of the studies. Of the 12 studies, only six reported on how ethical approval or consent of the participants was attained.

Of the nine qualitative studies, eight of them had an overall rating of good (++) according to the NICE qualitative appraisal tool. This rating meant that most of the checklist criteria had been fulfilled and where they had not been fulfilled the conclusions were very unlikely to alter.

Two out of the four mixed-methods studies scored 100% (****) on the MMAT appraisal tool. The other two scored 75% (***): studies by Kirby et al. (2009) and Oriol-Zerbe et al. (2007) did not consider how the findings related to the researcher’s influence in the interactions with the participants. A summary of the quality appraisal of all the included studies is available in Additional File 6 on Table S5-6*.*

### Factors influencing the implementation of cardiovascular risk scoring in primary care

**Fig. 2:**
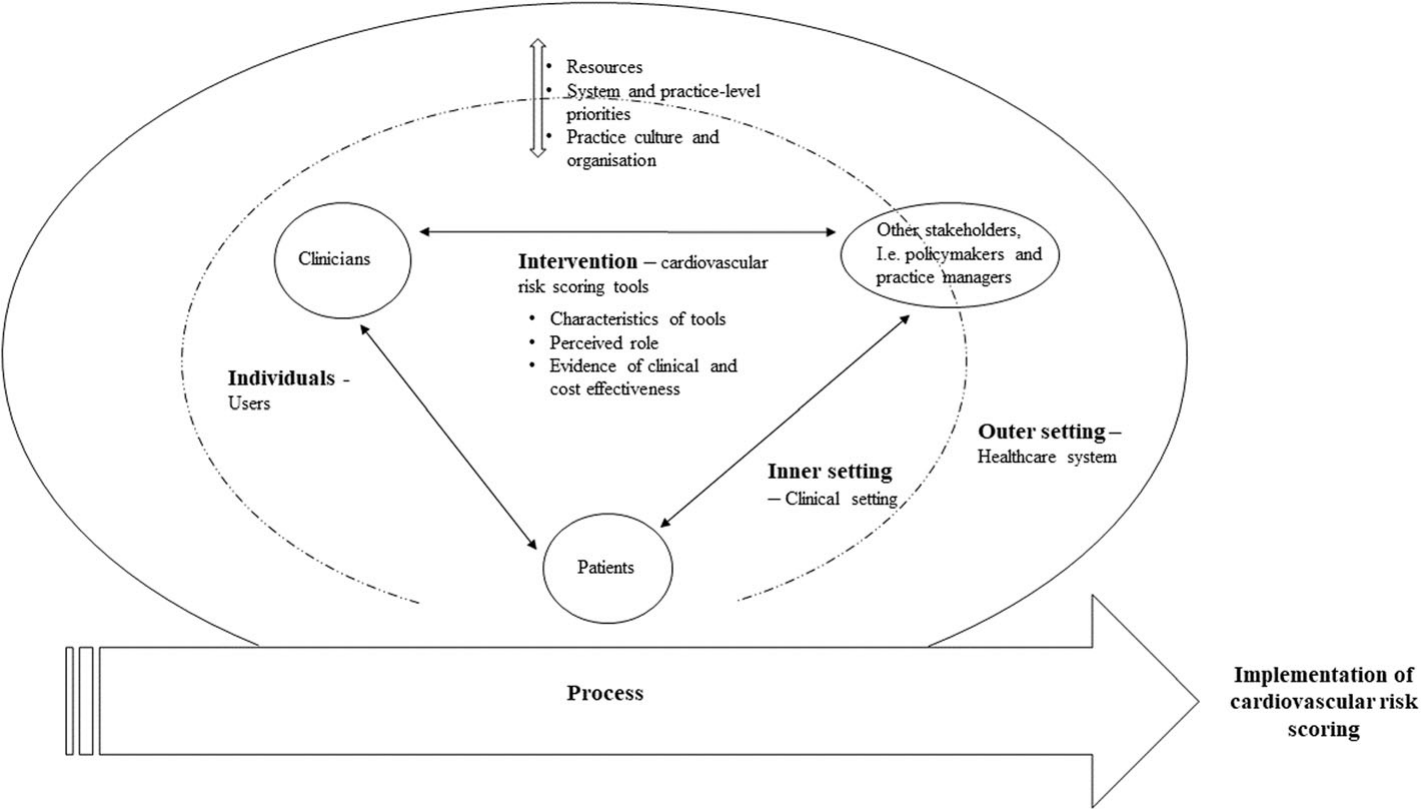
Factors influencing cardiovascular risk scoring in primary care settings

Three overarching constructs: the healthcare system and clinical setting, the users of cardiovascular risk scoring tools and cardiovascular risk scoring tools were identified (see Fig. 2). However, presenting these results in this format should not suggest that each construct and underlying factors exist in isolation. These constructs and underlying factors interact in rich and multifarious ways to influence implementation effectiveness. This review suggests that these factors have porous boundaries. For example, the knowledge and understanding of cardiovascular risk, cardiovascular disease and its management were a primary influence across these three overarching concepts.

We have presented these factors against the backdrop of the five domains of the CFIR (Fig. 2 and Table 3).

**Table 3:** Facilitators and barriers to cardiovascular risk scoring in primary care

CFIR domains involved	Factors influencing cardiovascular risk scoring in primary care		Facilitators	Barriers
Outer setting and inner setting process	Healthcare system and clinical setting	Resources	Adequately resourced healthcare systems with dedicated funding for the prevention of cardiovascular disease	Staffing shortages resulting in high workload, inadequate or no budgets for preventive services, lack of health information systems and lack of equipment to measure all the risk factors essential for risk scoring
		System and practice-level priorities	None reported	Prevention not prioritised for practice in health systems
		Practice culture and organisation	Supportive prevention care programs and pathways, task shifting, reallocation and sharing and having the appropriate individuals involved in prevention activities	Lack of interest and motivation to engage in preventive services, the practice of defensive medicine, a lack of collaboration between health workers and other staff and presence of disruptive professional hierarchies
Individual process	Users	Attributes of the users	*Patients:* knowledge and understanding of cardiovascular risk, disease and management, reinforcing personal circumstances and experiences, support from family, patients’demands and knowledge of their rights *Clinicians:* knowledge and understanding of cardiovascular risk, disease and management, knowledge of the benefits of cardiovascular risk scoring regarding patient care and therapeutic decision-making	*Patients:* complex patients (comorbidities and advanced age), patients who do not want to know their cardiovascular risk, negative perception of risk assessment, lack of knowledge on cardiovascular risk, disease and its management, patients’ health priorities, undermining personal circumstances, experiences and demands, surrounding environment, socioeconomic factors, fears and expectations of risk assessment *Clinicians:* negative perception of risk assessment, inadequate knowledge on cardiovascular risk, disease and its management, lack of knowledge on how to use cardiovascular risk scoring tools, lack of motivation to use these tools, difficulty in communicating cardiovascular risk to patients, challenges in communicating prevention and self-management aids to patients *Other stakeholders:* perceived interference and misuse of cardiovascular risk scoring as an intervention, lack of consensus on the use of risk scoring tools, general lack of interest, conflicting interests and poor tool reviewing processes
		Interaction between the users	A supportive and longstanding relationship between the clinician and patient	lack of communication and involvement in decision-making between clinicians and other stakeholders
Intervention process	Cardiovascular risk scoring tools	Characteristics of the tools	Easy to use, use of charts or calculators, presenting risk scores as colour codes instead of percentages and incorporating risk tools into clinical systems as programs or software	Outdates rapidly, time-consuming leading to prolonged consultations, lowers the quality of the consultation, does not include all the principal risk factors, the risk duration calculated is too long, it is complex to use and explain to patients, has technical problems and does not communicate with other programs
		The perceived role of the tools	Supportive role to clinical practice, i.e. to help understand risk, motivate patients, improve follow-up, educate patients and serve as a checklist for risk assessment	Over and underestimates risk leading to over or under treatment, it interferes with the clinicians’ decision-making process and it is less superior to clinical judgement
		Evidence of clinical and cost-effectiveness	Providing evidence that these tools were accurate in predicting risk, that they included the main risk factors for cardiovascular disease and that they led to better therapeutic decisions	It does not contribute to reducing healthcare costs. Unclear prediction rules were associated with prediction inaccuracies

### The healthcare system and clinical setting

The factors that emerged from this construct included resources, system and practice-level priorities, the practice culture of the clinical setting and how the practice was organised.

#### Resources

Physical and human resources acted as facilitators or barriers to cardiovascular risk scoring in primary care settings. An adequately resourced health system with a dedicated budget for the prevention of cardiovascular disease was viewed as a facilitator [40]. However, most studies regardless of setting reported the presence of inadequate resources to support cardiovascular risk assessment [41–46]. There was a lack of health information systems, lack of equipment to measure all the risk factors and a scarcity of human resources associated with staff shortages and high workload. The lack of health information systems and equipment was only reported in lower middle-income countries [46].

#### System and practice-level priorities

In healthcare systems where prevention of cardiovascular disease was not prioritised, cardiovascular risk scoring was implemented sub-optimally [40, 42, 46]. This was both in high and middle-income countries. However, for middle-income countries, the reasons given for not prioritising prevention were that the healthcare system was satisfying a higher demand for ‘more urgent’ social and health problems [42, 46]. This shifted the priority away from primary prevention and chronic condition management. Further, in some settings where prevention of cardiovascular disease was a priority, conflict was endemic in deciding to whom this mandate belonged, i.e. clinicians versus local authorities [36].

#### Practice culture and organisation

The adoption of cardiovascular risk scoring was seen in settings that had existing cardiovascular prevention care programs and pathways [40]. However, adequate organisation of these prevention activities was of importance, i.e. staffed by appropriate persons and proximity to the clinical settings for the clinician and the patient [35, 40].

Defensive medicine, a practice of recommending tests or treatment that are not necessarily the best option to the patient but as a function to protect the clinician against liability, was a barrier to cardiovascular risk scoring [36]. Another barrier was the presence of disruptive professional hierarchies [36]. Specialists or clinicians who ranked higher in practice were viewed in high regard even when they did not adhere to guidelines such as cardiovascular risk scoring. This sets a precedent for practice in the clinical setting, and junior clinicians did not feel empowered to question or disrupt. Also, the lack of collaboration between healthcare workers and administrative staff either due to increased workloads or a lack of communication was a barrier to cardiovascular risk scoring [46]. This practice of defensive medicine and the presence of disruptive professional hierarchies were only reported in high-income countries. On the other hand, the lack of collaboration between healthcare workers and administrative staff was only reported in lower middle-income countries.

### The users

These factors included the attributes of users (direct or indirect) and their interactions.

#### Attributes of the users

In this case, the review explores the characteristics of clinicians, patients and other stakeholders who influence the use of cardiovascular risk scoring. These characteristics acted as either facilitators or barriers in various settings. For both patients and clinicians, the perception and understanding of cardiovascular risk, disease and its management were a significant driver for cardiovascular risk scoring [33, 35, 36, 38, 40, 42, 44, 46–48]. In most studies and for both clinicians and patients, there was an indication that cardiovascular risk, its assessment and management was marred with negative perceptions unsupported by evidence. For example, both clinicians and patients reported the understanding that cardiovascular disease risk assessment focused only on an individual or single risk factor.

Personal circumstances and experiences of patients also influenced the adoption of cardiovascular risk scoring [40]. These circumstances and experiences included the environment in which the patients lived in, their socioeconomic standing, their fears, motivations and expectations of cardiovascular risk assessment and the support available [35, 36, 40, 42, 51]. Patients that lived in surroundings that were not conducive to making lifestyle behavioural adjustment were not keen on getting assessed for cardiovascular risk. Those who had a shared experience of loss of a family member or loved one through a cardiovascular event fatality reported being open to routine risk assessment. In lower middle-income countries, patients reported being unable to afford risk assessment procedures and consequent management—pharmacological and lifestyle adjustment [39, 42]. The patients’ priorities, which were either set by the patients or the clinicians, leaned towards what was considered ‘urgent for the patient’.

In many cases, cardiovascular risk scoring was not viewed as urgent enough in this hierarchy [40, 46, 49]. However, some patients demanded to be assessed for cardiovascular risk and this facilitated cardiovascular risk scoring [36, 47, 51]. Conversely, there were instances where patients demanded treatment regardless of their cardiovascular risk. This was a barrier to cardiovascular risk scoring [36, 42].

The lack of knowledge on how to use cardiovascular risk tools was unique to clinicians [41–43, 50, 52–55]. This was complicated by factors such as the lack of sufficient understanding of cardiovascular risk and disease and the role of cardiovascular risk scoring in informing management. Other complicating factors included patients who presented to the clinical settings with advanced age [39, 41, 43, 44, 52, 53, 56]. Clinicians also expressed difficulty in communicating cardiovascular risk scores to their patients after risk assessment. This was coupled with challenges in offering prevention and self-management support to patients [40]. There was also a general lack of motivation to use cardiovascular risk scoring tools [46]. However, knowledge about the tools and their benefits in clinical practice, such as improving care and bettering therapeutic decision making by clinicians, facilitated the use of these tools [33, 35, 38, 42, 44–47, 53, 56–58].

Stakeholders, i.e. ‘decision-makers’ who included policymakers and practice managers, were also reported to lack interest or having conflicting views regarding the prevention and management of cardiovascular risk [40, 41, 58, 59]. These conflicts involved whether prevention and management of cardiovascular risk was a priority, and whose priority it was in the clinical setting. They lacked consensus on the use of risk scoring tools and had unsatisfactory processes in place to continually review risk scoring tools used in practice [40, 45]. In lower middle-income countries, stakeholders were reported to have a disruptive influence on the prevention and management of cardiovascular risk. It was perceived that stakeholders in health authorities had the intention of ‘misusing’ tools used for cardiovascular risk scoring [39]. The meaning of the term ‘misusing’ was not clear from the data.

#### Interactions between the users

A supportive and longstanding relationship between the clinician and patient facilitated the use of cardiovascular risk scoring [51]. Clinicians felt that ‘floating’ patients already had a regular doctor to whom this responsibility of prevention belonged—the clinicians were only interested in dealing with the immediate complaint from the patient. The interaction between the clinicians and the other stakeholders was only at a policy level. There was no consensus on the use of these tools, partially explained by the lack of communication and involvement in decision making [40, 45].

### Cardiovascular risk scoring tools

These tools varied in complexity, design and packaging. For example, some of the tools were presented as risk tables, calculators or charts; some coded risk levels by colour or percentages and others were designed for specific countries or regions. It is important to note that close to half (*n* = 5) of these tools, though designed for specific geographical regions and populations, were often used outside these jurisdictions (Table 2).

The factors influencing cardiovascular risk scoring were linked to the characteristics of the tools, the perceived role of the tools in clinical practice and the evidence of efficiency and effectiveness.

#### Characteristics of the tools

The use of charts, calculators and colour codes was reported to facilitate cardiovascular risk scoring due to the perceived ease of use. Besides, having these tools incorporated into existing clinical systems as software or web-based applications was facilitative [35, 42, 57, 60]. However, some studies reported that these tools were complex to use, to explain to patients and that they had technical problems [37, 40, 47, 56]. For example, they were not communicating with other clinical support programs. Many of the studies, irrespective of the type of tool used, reported that these tools were time-consuming, leading to prolonged and low-quality consultations [34, 39, 41–45, 52, 54–57]. Other studies pointed out that the tools outdated rapidly and consequently did not include all the important risk factors needed to assess for cardiovascular risk [36, 46, 53].

#### The perceived role of the tools

Similarly, the perceived role of these tools in clinical practice was both a facilitator and barrier. For example, clinicians who used the tools saw it as helpful in understanding risk, motivating patients, improving follow-up, educating patients and as a checklist for risk assessment [33, 42, 43, 45, 49, 61–64]. However, other clinicians viewed cardiovascular risk scoring as an intervention that over or/and underestimated risk leading to therapeutic inaccuracies [39, 44, 45]. These tools were also perceived as interfering with the clinician’s decision-making process [39, 44, 45, 56]. This, perhaps, is about clinicians considering these tools to be less superior to their clinical judgement [45, 53, 65, 66].

#### Evidence of clinical and cost-effectiveness

Unclear prediction rules were a barrier to using cardiovascular risk scoring tools, as this was associated with prediction inaccuracies [40, 42, 44–46, 53, 61). However, providing evidence that these tools were accurate in predicting risk, that they included the main risk factors for cardiovascular disease and that they led to better therapeutic decisions was facilitative [33, 42, 43, 45, 49, 61–63]. Lastly, in some studies, there was an uncertainty on the role of cardiovascular risk scoring in reducing health costs in healthcare systems [39, 44]. This, too, influenced use and implementation both at a policy and practice level.

A summary of these factors influencing cardiovascular risk scoring in primary care, categorised as facilitators or barriers, is presented in Table 3.

### A summary of additional analysis

#### Variations across Gross National Income classification

Many of the factors influencing cardiovascular risk scoring were similar across Gross National Income classification. Nevertheless, there were two salient barriers reported only in lower middle-income countries: the disruptive influence of stakeholders and the unaffordability of some of the assessments essential to risk scoring by patients.

#### Variations between patients and clinicians

For both clinicians and patients, knowledge and understanding of cardiovascular risk, cardiovascular disease and its management were a facilitator and barrier to cardiovascular risk scoring. However, some factors were specific to either patients or clinicians. The factors attributed to patients were more assorted in that there were not confined to the clinical setting or practice as was in the clinicians’ case. In patients, these factors included their circumstances and experiences, familial relations, awareness of their rights to health, their priorities, socioeconomic factors, emotions and their relationship with the clinician. As for clinicians, these included the perceived role of the risk scoring tools in the context of their practice and their abilities in delivering care.

#### Variations across physicians, non-physicians and other stakeholders

For physicians and non-physicians such as nurses, the facilitators and barriers to cardiovascular risk scoring were similar and spanned through the overarching concepts of cardiovascular risk scoring tools, users, the clinical setting and the healthcare system*.* As for other stakeholders, two salient barriers were reported: the lack of collaboration between clinicians and non-clinicians and not prioritising prevention and management of cardiovascular risk in the clinical setting and health care system.

### Confidence/certainty of the review findings

Table S8 in Additional File 7 presents a summary of the qualitative findings with their CERQual evidence profile and confidence assessments. All the findings in this synthesis were assessed as being of moderate or high confidence. The attributes of the users and the characteristics of the tools were assessed as being high in confidence.

## Discussion

### Summary and interpretation of findings

This synthesis aimed to synthesise the current knowledge of the factors influencing the implementation of cardiovascular risk scoring as a cardiovascular disease prevention strategy in primary care settings. This synthesis broadly conceptualises these factors into those relating to cardiovascular risk tools, users, clinical setting and healthcare system.

This review adds to the understanding that the implementation of an intervention is affected by its interaction with the real world—users and the environment in which it is being used [67]. Adequately, the review shows that the factors influencing cardiovascular risk scoring are intricately related, and that no single factor is identifiable as a more influential facilitator or barrier. For example, not prioritising prevention in a healthcare system may inform how resources are allocated in these settings. At the user level, the users’ attributes, i.e. their perceptions about cardiovascular risk scoring, may inform their interaction with the tools and in turn, influence adoption. These perceptions may be informed by the users’ knowledge, skill levels, personal circumstances, experiences, socioeconomic status, fear and expectations. An important note was the elucidation that the users of an intervention comprise of various people who can be classified as either having a direct or indirect interaction with the tool—i.e. clinicians, patients and other stakeholders. These users are equally important in understanding who to engage when implementing an intervention.

The review also centres the importance of tool design and quality in the implementation process. The tool’s assembly and presentation have to be appropriate for its users. Notably, there was a lack of research from low income countries which might suggest limited adoption of CVD risk scoring. This finding may surmise the suboptimal use of these tools in these settings, as reported in a global survey conducted by the WHO in 2015 [16]. Further, the findings of this review support the use of the CFIR as a priori framework for implementation driven research. However, one new theme emerged from the data, the knowledge and belief about disease and risk*,* which we classified under the characteristics of individuals’ domain. This succinctly brings out the important difference between individuals having sufficient knowledge about an intervention from that of having knowledge of the disease and the accompanying components of the disease, such as its aetiology and risk factors.

### Findings in context

The findings of this review mirror the understanding that there are numerous obstacles to the implementation of targeted risk reduction interventions. In particular, other studies have broadly highlighted these obstacles also to include patients, clinicians, healthcare organisation, socioeconomic barriers and policies [68]. These barriers to implementation may partly explain the common suboptimal control of cardiovascular risk factors, especially in high-risk patients [69–71].

Various forms of resources, i.e. fiscal and human, are vital in the implementation process [72]. Scarcity in either leads to the disruptive practice of prioritising curative care over prevention [73]. Consequently, essential tasks such as educating patients on risk and self-management have taken a back seat. Efforts to counter this inadequacy have been successful through innovative practices such as task sharing and shifting [74, 75]. Some countries have recognised community health workers as principal and resourceful caregivers, placing them at the forefront of prevention efforts in the communities [76–78]. However, this should not deter from the central fact that healthcare systems should intentionally focus on building structural and human infrastructure in support of preventive medicine in primary care settings. Having supportive programs and pathways such as health information systems in clinical practice has been shown to support risk scoring in the prevention of cardiovascular disease [79, 80]. Conversely, in acknowledging the complexity that healthcare systems posit, some findings have also shown that adequately resourced systems do not necessarily lead to positive implementation outcomes. This aptly brings out the complex nature of implementation processes [81].

Users’ attributes, i.e. their knowledge, skills and perceptions, are seen to play a vital role in realising cardiovascular risk scoring in this review. This is not surprising as many other studies have reported that primary care health workers often report that they lack the skills to manage cardiovascular risk [82, 83]. A patients’ understanding of cardiovascular risk has been associated with socioeconomic status and the level of education [84, 85]. There is a positive association between the level of knowledge on cardiovascular disease and its risk with the use of cardiovascular risk scores [86]. However, it is generally reported that clinicians find it challenging to communicate risk to their patients [87–91]. The communication of cardiovascular risk is no different [40]. This notwithstanding that risk scoring tools have been credited with assisting clinicians in better relaying risk messages [92–94]. Communicating risk to patients has been lauded in improving cardiovascular risk outcomes in asymptomatic patients [95].

The role of risk scoring in clinical practice is an ongoing debate. Many studies in this review report that the use of risk scores is time-consuming, leading to prolonged consultations and consequently, lowering the quality of consultation. However, numerous studies have shown that the general use of personalised risk estimates adds value by improving patients’ satisfaction and increasing the intention to change behaviour [96–98]. It is unclear the role that clinical judgement takes in the use of risk scoring tools with many clinicians choosing to regard these tools as less superior to their clinical judgement. However, evidence from various studies has shown that there is a significant mismatch between clinicians’ estimated cardiovascular risk and the risk assessed using risk scoring tools, especially in high-risk patients [66, 83, 99–101]. This, then, necessitates the need for the existing assessment and management algorithms for cardiovascular disease [102–104]. Equally, although these guidelines are known to clinicians, studies have shown that they are consistently not implemented in practice [105]. Perhaps, because understanding probabilistic risk information does not necessarily influence the clinician’s decision making or practice [106].

The efficacy of cardiovascular risk scoring tools in systematic reviews shows that providing cardiovascular scores is beneficial in reducing the levels of cardiovascular risk factors [1, 6]. However, there is still no evidence showing that the use of these tools influences outcomes on mortality. This has been a significant barrier in influencing the adoption of these tools by clinicians. Some of the challenges of showing association between the use of these tools and terminal outcomes such as mortality may be tied to the challenges of implementation [6]. Issues of the technical complexity of the risk scoring tools have been addressed through changes in design and packaging—the use of charts and tables in settings without computer connectivity and the use of embedded software programs in settings with existing electronic health information systems [56, 107]. However, there is a need for frequent training and retraining to keep up with the dynamism of these tools [108].

While acknowledging that healthcare systems vary greatly, one commonality of these systems is striving to deliver affordable, cost-effective and good quality care. The unaffordability of some of the laboratory-based investigations used in cardiovascular risk scoring has been emphasised in resource-limited healthcare systems, predominantly in LMICs, by patients [39]. However, organisations such as the WHO have provided affordable solutions by simplifying risk assessment and management in these settings through tailor-made packages [107, 109, 110].

### Strengths and limitations

This review is strengthened by its methodology, a design that adequately addresses the burgeoning gap between research and practice by providing actionable outcomes of direct relevance to policy and practice [111]. While an exhaustive search was undertaken, it is possible that some primary studies were missed. Most of the studies (80%) included in this review are from high-income countries. Very few studies are from low and middle-income countries. Therefore, caution should be taken when attempting to apply these findings in our study to these settings. Lastly, mixed-methods systematic reviews of this kind are an emerging methodology and consequently, have limited methodological literature [25].

### Implications for research and practice

While we found a small but good quality evidence-based on current knowledge of the factors influencing the implementation of cardiovascular risk scoring in primary care settings, these are limited to high income countries, and targeted research is needed in low and middle-income countries. Across settings, there remains the need for high-quality evidence for the prospective use of cardiovascular risk scoring tools on health outcomes. This could take the form of effectiveness-implementation hybrid designs combining the elements of clinical effectiveness and implementation research to enhance public health impact.

## Conclusion

This review reinforces the understanding that implementation processes are complex and for favourable implementation outcomes: acceptability, adoption, appropriateness, feasibility, fidelity, implementation cost, coverage and sustainability—policymakers and implementation practitioners must be willing to engage with the various rudiments put forward in this review. These rudiments involve having enough understanding of the intervention, the environment, the users and their various interactions with all the above. While these findings bolster the understanding of implementation complexity, there exists limited research in the context of low and middle-income countries. Notwithstanding the need to direct resources in bridging this gap, it is also crucial that these efforts are in concert with providing high-quality evidence on the clinical effectiveness of using cardiovascular risk scoring to improve cardiovascular disease outcomes of mortality and morbidity.

## Supplementary information

**Additional File 1: Table S1.** PRISMA Checklist

**Additional File 2:.** Search Terms and Strategy for Databases

**Additional File 3: Table S2.** Characteristics of Included studies | **Table S3.** Characteristics of Excluded studies and Reasons for exclusion

**Additional File 4: Figure S1**. PRISMA Flowchart

**Additional File 5: Table S4.** Summary of Facilitators and Barriers as coded on the CFIR

**Additional File 6: Table S5-6.** Quality Appraisal for Included studies

**Additional File 7: Table S7.** Summary of the qualitative findings with their CERQual evidence profile and confidence assessments

## Data Availability

All data generated or analysed during this study are included in this published article and its supplementary information files.

## Abbreviations

ACC: American College of Cardiology
AHA: American Heart Association
BeHEMoTh: Behaviour of interest, Health context, Exclusions and Models and Theories
CERQual: Confidence in the Evidence from Reviews of Qualitative research
CVD: Cardiovascular disease
CFIR: Consolidated Framework for Implementation Research
CINAHL: Cumulative Index to Nursing and Allied Health Literature
EMBASE: Excerpta Medica dataBASE
ENTREQ: Enhancing Transparency in Reporting the Synthesis of Qualitative research
MEDLINE: Medical Literature Analysis and Retrieval System Online
MMAT: Mixed Methods Appraisal Tool
NICE: National Institute for Health and Care Excellence
NCD: Non-communicable disease
PICO: Population, Intervention, Comparator, Outcome
PRISMA-P: Preferred Reporting Items for Systematic review and Meta-Analysis Protocol
PROCAM: Prospective Cardiovascular Munster
PsycINFO: Database of abstracts of literature in the field of psychology
QRISK: Prediction algorithm for cardiovascular disease using risk factors
SCORE: European cardiovascular disease risk assessment model
WHO: World Health Organisation
