# Supplementary material for: Factors influencing the implementation of cardiovascular risk scoring in primary care: a mixed-method systematic review
Source: Implement Sci. 2020 Jul 20;15:57. doi: 10.1186/s13012-020-01022-x (PMC7370418; doi:10.1186/s13012-020-01022-x)
Supplement: Supplementary file 2 — Additional File 2:. Search Terms and Strategy for Databases [file 13012_2020_1022_MOESM2_ESM.docx]

**Search Terms and Strategy for Databases**

| [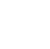](http://ezproxy-prd.bodleian.ox.ac.uk:2081/sp-3.28.0a/ovidweb.cgi?&S=CKKCFPKELJDDKCHKNCFKKAIBCKGJAA00&R=31&Search+Annotations+Options=S)[# ▲](http://ezproxy-prd.bodleian.ox.ac.uk:2081/sp-3.28.0a/ovidweb.cgi?&S=CKKCFPKELJDDKCHKNCFKKAIBCKGJAA00&Sort+Sets=descending) | Searches **- Medline** | **Results** |
| --- | --- | --- |
|  |  |  |
| 1 | *cardiovascular diseases/ or *heart diseases/ or *vascular diseases/ | 161316 |
| 2 | cardiovascular diseases/pc or heart diseases/pc or vascular diseases/pc | 34802 |
| 3 | (((cardiovavascular or cardio-vascular or heart or vascular) adj (risk? or disease* or health)) or cvd).ti. | 70553 |
| 4 | 1 or 2 or 3 | 226915 |
| 5 | Risk Management/mt, st, ut [Methods, Standards, Utilization] | 4314 |
| 6 | Risk Assessment/mt, st, ut | 27460 |
| 7 | *Risk Assessment/ | 24591 |
| 8 | Primary Prevention/ and (Risk Management/ or Risk Assessment/ or Risk Factors/) | 6231 |
| 9 | (risk? adj3 (scor* or algorithm? or table? or model* or strateg* or engine? or tool? or index or indices or chart?)).ti,ab. | 64688 |
| 10 | (risk? adj3 (assess* or estimat* or calculat* or reduc* or stratif*)).ti,ab. | 279112 |
| 11 | 5 or 6 or 7 or 8 or 9 or 10 | 348261 |
| 12 | Ambulatory Care/ | 40068 |
| 13 | exp Ambulatory Care Facilities/ | 51355 |
| 14 | general practice/ or family practice/ | 71757 |
| 15 | general practitioners/ or physicians, family/ or physicians, primary care/ or physicians/ | 104504 |
| 16 | Primary Health Care/ | 67426 |
| 17 | Office Visits/ | 6400 |
| 18 | (ambulatory adj3 (care or setting? or facilit* or ward? or department? or service?)).ti,ab. | 15593 |
| 19 | (((general or family) adj2 (practi* or doctor?)) or physician?).ti,ab. | 426348 |
| 20 | (primary care or primary health care or primary healthcare).ti,ab. | 114394 |
| 21 | (after hour? or afterhour? or "out of hour?" or ooh).ti,ab. | 4134 |
| 22 | (clinic? or visit?).ti,ab. | 424796 |
| 23 | ((health* or medical) adj2 (center? or centre?)).ti,ab. | 107616 |
| 24 | Community Health Services/ | 29742 |
| 25 | Community Health Workers/ | 4395 |
| 26 | (community adj3 (service? or worker? or volunteer? or aide? or assistant?)).ti,ab. | 20250 |
| 27 | (health adj2 (worker? or volunteer? or aide? or assistant?)).ti,ab. | 31496 |
| 28 | 12 or 13 or 14 or 15 or 16 or 17 or 18 or 19 or 20 or 21 or 22 or 23 or 24 or 25 or 26 or 27 | 1139877 |
| 29 | Practice Patterns, Physicians'/ | 51924 |
| 30 | Health Knowledge, Attitudes, Practice/ or Attitude of Health Personnel/ | 193726 |
| 31 | Guideline Adherence/ | 27996 |
| 32 | (barrier? or obstacle? or challeng*).ti,ab. | 899044 |
| 33 | (facilitat* or enabl* or opportunit* or influence* or motivat*).ti,ab. | 2187847 |
| 34 | (implement* or uptake or util?e? or utili?ation or adopt*).ti,ab. | 1033524 |
| 35 | (attitud* or perception* or perspective* or view* or opinion* or expectation? or fear?).ti,ab. | 1107127 |
| 36 | (knowledge or understand* or comprehen* or aware* or communicat*).ti,ab. | 1976260 |
| 37 | 29 or 30 or 31 or 32 or 33 or 34 or 35 or 36 | 5794691 |
| 38 | 4 and 11 and 28 and 37 | 1411 |

| # | Searches **- Embase** | **Results** |
| --- | --- | --- |
| 1 | *cardiovascular diseases/ or *heart diseases/ or *vascular diseases/ | 43877 |
| 2 | cardiovascular diseases/pc or heart diseases/pc or vascular diseases/pc | 7677 |
| 3 | exp *cardiovascular risk/ | 34587 |
| 4 | (((cardiovavascular or cardio-vascular or heart or vascular) adj (risk? or disease* or health)) or cvd).ti. | 84974 |
| 5 | 1 or 2 or 3 or 4 | 159442 |
| 6 | *Risk Management/ | 15511 |
| 7 | *Risk Assessment/ | 40299 |
| 8 | Primary Prevention/ and (Risk Management/ or Risk Assessment/ or Risk Factors/) | 8817 |
| 9 | (risk? adj3 (scor* or algorithm? or table? or model* or strateg* or engine? or tool? or index or indices or chart?)).ti,ab. | 105059 |
| 10 | (risk? adj3 (assess* or estimat* or calculat* or reduc* or stratif*)).ti,ab. | 398005 |
| 11 | 6 or 7 or 8 or 9 or 10 | 498352 |
| 12 | Ambulatory Care/ | 35223 |
| 13 | general practice/ | 77978 |
| 14 | general practitioner/ or physician/ | 330789 |
| 15 | Primary Health Care/ or primary medical care/ | 145000 |
| 16 | (ambulatory adj3 (care or setting? or facilit* or ward? or department? or service?)).ti,ab. | 20749 |
| 17 | (((general or family) adj2 (practi* or doctor?)) or physician?).ti,ab. | 576576 |
| 18 | (primary care or primary health care or primary healthcare).ti,ab. | 148440 |
| 19 | (after hour? or afterhour? or "out of hour?" or ooh).ti,ab. | 5879 |
| 20 | (clinic? or visit?).ti,ab. | 655465 |
| 21 | ((health* or medical) adj2 (center? or centre?)).ti,ab. | 152214 |
| 22 | health center/ | 29481 |
| 23 | health auxiliary/ | 5330 |
| 24 | (community adj3 (service? or worker? or volunteer? or aide? or assistant?)).ti,ab. | 25328 |
| 25 | (health adj2 (worker? or volunteer? or aide? or assistant?)).ti,ab. | 36670 |
| 26 | 12 or 13 or 14 or 15 or 16 or 17 or 18 or 19 or 20 or 21 or 22 or 23 or 24 or 25 | 1597532 |
| 27 | health personnel attitude/ or physician attitude/ or protocol compliance/ | 131055 |
| 28 | (barrier? or obstacle? or challeng*).ti,ab. | 1111742 |
| 29 | (facilitat* or enabl* or opportunit* or influence* or motivat*).ti,ab. | 2658739 |
| 30 | (implement* or uptake or util?e? or utili?ation or adopt*).ti,ab. | 1301508 |
| 31 | (attitud* or perception* or perspective* or view* or opinion* or expectation? or fear?).ti,ab. | 1371243 |
| 32 | (knowledge or understand* or comprehen* or aware* or communicat*).ti,ab. | 2463078 |
| 33 | 27 or 28 or 29 or 30 or 31 or 32 | 7067677 |
| 34 | 5 and 11 and 26 and 33 | 1293 |

| [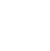](http://ezproxy-prd.bodleian.ox.ac.uk:2081/sp-3.28.0a/ovidweb.cgi?&S=CKKCFPKELJDDKCHKNCFKKAIBCKGJAA00&R=11&Search+Annotations+Options=S)[# ▲](http://ezproxy-prd.bodleian.ox.ac.uk:2081/sp-3.28.0a/ovidweb.cgi?&S=CKKCFPKELJDDKCHKNCFKKAIBCKGJAA00&Sort+Sets=descending) | Searches **- PsycINFO** | **Results** |
| --- | --- | --- |
| 1 | Cardiovascular disorders/ | 8914 |
| 2 | (((cardiovavascular or cardio-vascular or heart or vascular) adj (risk? or disease* or health)) or cvd).ti. | 3033 |
| 3 | 1 or 2 | 11378 |
| 4 | Risk Management/ | 4723 |
| 5 | Risk Assessment/ | 12447 |
| 6 | Prevention/ and (Risk Management/ or Risk Assessment/ or Risk Factors/) | 2470 |
| 7 | (risk? adj3 (scor* or algorithm? or table? or model* or strateg* or engine? or tool? or index or indices or chart?)).ti,ab. | 12025 |
| 8 | (risk? adj3 (assess* or estimat* or calculat* or reduc* or stratif*)).ti,ab. | 39965 |
| 9 | 4 or 5 or 6 or 7 or 8 | 57504 |
| 10 | physicians/ or family physicians/ or general practitioners/ | 26405 |
| 11 | Primary Health Care/ | 16583 |
| 12 | (ambulatory adj3 (care or setting? or facilit* or ward? or department? or service?)).ti,ab. | 1954 |
| 13 | (((general or family) adj2 (practi* or doctor?)) or physician?).ti,ab. | 70964 |
| 14 | (primary care or primary health care or primary healthcare).ti,ab. | 29812 |
| 15 | (after hour? or afterhour? or "out of hour?" or ooh).ti,ab. | 408 |
| 16 | (clinic? or visit?).ti,ab. | 84611 |
| 17 | ((health* or medical) adj2 (center? or centre?)).ti,ab. | 18681 |
| 18 | (community adj3 (service? or worker? or volunteer? or aide? or assistant?)).ti,ab. | 13638 |
| 19 | (health adj2 (worker? or volunteer? or aide? or assistant?)).ti,ab. | 7859 |
| 20 | 10 or 11 or 12 or 13 or 14 or 15 or 16 or 17 or 18 or 19 | 199427 |
| 21 | health personnel attitudes/ | 17762 |
| 22 | (barrier? or obstacle? or challeng*).ti,ab. | 270221 |
| 23 | (facilitat* or enabl* or opportunit* or influence* or motivat*).ti,ab. | 775991 |
| 24 | (implement* or uptake or util?e? or utili?ation or adopt*).ti,ab. | 259587 |
| 25 | (attitud* or perception* or perspective* or view* or opinion* or expectation? or fear?).ti,ab. | 908229 |
| 26 | (knowledge or understand* or comprehen* or aware* or communicat*).ti,ab. | 926641 |
| 27 | 21 or 22 or 23 or 24 or 25 or 26 | 2121600 |
| 28 | 3 and 9 and 20 and 27 | 163 |

| [# ▲](http://ezproxy-prd.bodleian.ox.ac.uk:2081/sp-3.28.0a/ovidweb.cgi?&S=CKKCFPKELJDDKCHKNCFKKAIBCKGJAA00&Sort+Sets=descending) | Searches **– Global Health** | **Results** |
| --- | --- | --- |
|  |  |  |
| 1 | exp Cardiovascular Diseases/ | 100004 |
| 2 | (((cardiovavascular or cardio-vascular or heart or vascular) adj (risk? or disease* or health)) or cvd).ti. | 7986 |
| 3 | 1 or 2 | 100487 |
| 4 | Risk Analysis/ | 2078 |
| 5 | Risk Assessment/ | 42333 |
| 6 | Disease Prevention/ and Risk Factors/ | 7077 |
| 7 | (risk? adj3 (scor* or algorithm? or table? or model* or strateg* or engine? or tool? or index or indices or chart?)).ti,ab. | 15743 |
| 8 | (risk? adj3 (assess* or estimat* or calculat* or reduc* or stratif*)).ti,ab. | 77698 |
| 9 | 4 or 5 or 6 or 7 or 8 | 115525 |
| 10 | physicians/ or general practitioners/ | 18267 |
| 11 | Primary Health Care.sh. | 10286 |
| 12 | (ambulatory adj3 (care or setting? or facilit* or ward? or department? or service?)).ti,ab. | 1556 |
| 13 | (((general or family) adj2 (practi* or doctor?)) or physician?).ti,ab. | 41875 |
| 14 | (primary care or primary health care or primary healthcare).ti,ab. | 21898 |
| 15 | (after hour? or afterhour? or "out of hour?" or ooh).ti,ab. | 302 |
| 16 | exp health centres/ or Community Health Services/ | 11893 |
| 17 | (clinic? or visit?).ti,ab. | 80670 |
| 18 | ((health* or medical) adj2 (center? or centre?)).ti,ab. | 25171 |
| 19 | community health workers/ | 229 |
| 20 | (community adj3 (service? or worker? or volunteer? or aide? or assistant?)).ti,ab. | 6100 |
| 21 | (health adj2 (worker? or volunteer? or aide? or assistant?)).ti,ab. | 15527 |
| 22 | 10 or 11 or 12 or 13 or 14 or 15 or 17 or 18 or 20 or 21 | 165959 |
| 23 | attitudes/ | 41917 |
| 24 | (barrier? or obstacle? or challeng*).ti,ab. | 130295 |
| 25 | (facilitat* or enabl* or opportunit* or influence* or motivat*).ti,ab. | 284795 |
| 26 | (implement* or uptake or util?e? or utili?ation or adopt*).ti,ab. | 180439 |
| 27 | (attitud* or perception* or perspective* or view* or opinion* or expectation? or fear?).ti,ab. | 149051 |
| 28 | (knowledge or understand* or comprehen* or aware* or communicat*).ti,ab. | 287083 |
| 29 | 23 or 24 or 25 or 26 or 27 or 28 | 783509 |
| 30 | 3 and 9 and 22 and 29 | 596 |

| # | Query - **CINAHL** | **Results** |
| --- | --- | --- |
| S22 | S4 AND S9 AND S16 AND S21 | 277 |
| S21 | S17 OR S18 OR S19 OR S20 | 889,216 |
| S20 | TI ( (barrier* or obstacle* or challeng*) ) OR AB ( (barrier* or obstacle* or challeng*) ) OR TI ( (facilitat* or enabl* or opportunit* or influence* or motivat*) ) OR AB ( (facilitat* or enabl* or opportunit* or influence* or motivat*) ) OR TI ( (implement* or uptake or utilis* or utiliz* or adopt*) ) OR AB ( (implement* or uptake or utilis* or utiliz* or adopt*) ) OR TI ( (attitud* or perception* or perspective* or view* or opinion* or expectatio*? or fear*) ) OR AB ( (attitud* or perception* or perspective* or view* or opinion* or expectatio*? or fear*) ) OR TI ( (knowledge or understand* or comprehen* or aware* or communicat*) ) OR AB ( (knowledge or understand* or comprehen* or aware* or communicat*) ) | 854,092 |
| S19 | (MH "Program Implementation") | 16,210 |
| S18 | (MH "Guideline Adherence") | 6,099 |
| S17 | (MH "Practice Patterns") OR (MH "Attitude of Health Personnel+") | 64,507 |
| S16 | S10 OR S11 OR S12 OR S13 OR S14 OR S15 | 335,836 |
| S15 | TI ( ((community N3 (service* or worker* or volunteer* or aide* or assistant*)) ) OR AB ( (community N3 (service* or worker* or volunteer* or aide* or assistant*)) ) OR TI ( ((health N3 (service* or worker* or volunteer* or aide* or assistant*)) ) OR AB ( ((health N3 (service* or worker* or volunteer* or aide* or assistant*)) ) | 66,822 |
| S14 | (MH "Community Health Services") OR (MH "Community Health Workers") | 15,016 |
| S13 | TI ( (ambulatory N3 (care or setting* or facilit* or ward* or department* or service*)) ) OR AB ( (ambulatory N3 (care or setting* or facilit* or ward* or department* or service*)) ) OR TI ( (((general or family) N2 (practi* or doctor*)) or physician*) ) OR AB ( (((general or family) N2 (practi* or doctor*)) or physician*) ) OR TI ( ("primary care" or "primary health care" or "primary healthcare") ) OR AB ( ("primary care" or "primary health care" or "primary healthcare") ) OR TI ( ("after hour*" or afterhour* or "out of hour*" or ooh) ) OR AB ( ("after hour*" or afterhour* or "out of hour*" or ooh) ) OR TI ( clinic OR clinics OR visit OR visits ) OR AB ( clinic OR clinics OR visit OR visits ) OR TI ( ((health* or medical) N2 (center* or centre*)) ) OR AB ( ((health* or medical) N2 (center* or centre*)) ) | 223,435 |
| S12 | (MH "Primary Health Care") OR (MH "Office Visits") | 40,307 |
| S11 | (MH "Family Practice") OR (MH "Physicians") OR (MH "Physicians, Family") | 59,056 |
| S10 | (MH "Ambulatory Care") OR (MH "Ambulatory Care Facilities") | 10,921 |
| S9 | S5 OR S6 OR S7 OR S8 | 76,241 |
| S8 | TI ( (risk* N3 (scor* or algorithm* or table* or model* or strateg* or engine* or tool* or index or indices or chart*)) ) OR TI ( (risk* N3 (scor* or algorithm* or table* or model* or strateg* or engine* or tool* or index or indices or chart*))(risk* N3 (assess* or estimat* or calculat* or reduc* or stratif*)) ) OR AB ( (risk* N3 (scor* or algorithm* or table* or model* or strateg* or engine* or tool* or index or indices or chart*)) ) OR AB ( (risk* N3 (assess* or estimat* or calculat* or reduc* or stratif*)) ) | 62,214 |
| S7 | (MH "Risk Management/MT/ST/UT") OR (MH "Risk Assessment/MT/ST/UT") | 5,545 |
| S6 | (MM "Risk Assessment") | 11,597 |
| S5 | (MM "Risk Management") | 5,657 |
| S4 | S1 OR S2 OR S3 | 37,982 |
| S3 | TI (((cardiovavascular or cardio-vascular or heart or vascular) N1 (risk* or disease* or health)) or cvd) | 12,823 |
| S2 | (MH "Cardiovascular Diseases/PC") OR (MH "Heart Diseases/PC") OR (MH "Vascular Diseases/PC") | 10,903 |
| S1 | (MM "Cardiovascular Diseases") OR (MM "Heart Diseases") OR (MM "Vascular Diseases") | 24,538 |

**Web of Science**

| # 5 | [289](http://apps.webofknowledge.com/summary.do?product=WOS&doc=1&qid=12&SID=E1FG8cKEIITJdtyTomF&search_mode=CombineSearches&update_back2search_link_param=yes) | #4 AND #3 AND #2 AND #1 |
| --- | --- | --- |
| # 4 | [11,867,108](http://apps.webofknowledge.com/summary.do?product=WOS&doc=1&qid=9&SID=E1FG8cKEIITJdtyTomF&search_mode=GeneralSearch&update_back2search_link_param=yes) | **TOPIC:** ((barrier* or obstacle* or challeng*)) *OR* **TOPIC:** ((facilitat* or enabl* or opportunit* or influence* or motivat*)) *OR* **TOPIC:** ((implement* or uptake or utilis* or utiliz* or adopt*)) *OR* **TOPIC:** ((attitud* or perception* or perspective* or view* or opinion* or expectatio*? or fear*)) *OR* **TOPIC:** ((knowledge or understand* or comprehen* or aware* or communicat*)) |
| # 3 | [921,764](http://apps.webofknowledge.com/summary.do?product=WOS&doc=1&qid=8&SID=E1FG8cKEIITJdtyTomF&search_mode=GeneralSearch&update_back2search_link_param=yes) | **TOPIC:** ((ambulatory NEAR/3 (care or setting* or facilit* or ward* or department* or service*))) *OR* **TOPIC:** ((((general or family) NEAR/2 (practi* or doctor*)) or physician*)) *OR* **TOPIC:** (("primary care" or "primary health care" or "primary healthcare")) *OR* **TOPIC:** (("after hour*" or afterhour* or "out of hour*" or ooh)) *OR* **TOPIC:** (clinic OR clinics OR visit OR visits) *OR* **TOPIC:** (((health* or medical) NEAR/2 (center* or centre*))) *OR* **TOPIC:** ((community NEAR/3 (service* or worker* or volunteer* or aide* or aessistant*))) *OR* **TOPIC:** ((health NEAR/2 (service* or worker* or volunteer* or aide* or assistant*))) |
| # 2 | [116,169](http://apps.webofknowledge.com/summary.do?product=WOS&doc=1&qid=7&SID=E1FG8cKEIITJdtyTomF&search_mode=GeneralSearch&update_back2search_link_param=yes) | **TOPIC:** ((risk* NEAR/3 (scor* or algorithm* or table* or model* or strateg* or engine* or tool* or index or indices or chart*))) *OR* **TOPIC:** ((risk* NEAR/3 (scor* or algorithm* or table* or model* or strateg* or engine* or tool* or index or indices or chart*))) |
| # 1 | [134,774](http://apps.webofknowledge.com/summary.do?product=WOS&doc=1&qid=11&SID=E1FG8cKEIITJdtyTomF&search_mode=AdvancedSearch&update_back2search_link_param=yes) | TS=((((cardiovavascular or cardio-vascular or heart or vascular) N (risk* or disease* or health)) or cvd)) |

**Cochrane**

| ID | Search - |
| --- | --- |
| #1 | MeSH descriptor: [Cardiovascular Diseases] this term only |
| #2 | MeSH descriptor: [Heart Diseases] this term only |
| #3 | MeSH descriptor: [Vascular Diseases] this term only |
| #4 | (((cardiovavascular or cardio-vascular or heart or vascular) near/2 (risk* or disease* or health)) or cvd):ti,ab,kw (Word variations have been searched) |
| #5 | #1 or #2 or #3 or #4 |
| #6 | MeSH descriptor: [Risk Management] this term only |
| #7 | MeSH descriptor: [Risk Assessment] this term only |
| #8 | MeSH descriptor: [Primary Prevention] this term only |
| #9 | (risk* near/3 (scor* or algorithm* or table* or model* or strateg* or engine* or tool* or index or indices or chart*)):ti,ab,kw or (risk* near/3 (assess* or estimat* or calculat* or reduc* or stratif*)):ti,ab,kw (Word variations have been searched) |
| #10 | #6 or #7 or #8 or #9 |
| #11 | MeSH descriptor: [Ambulatory Care] this term only |
| #12 | MeSH descriptor: [Ambulatory Care Facilities] explode all trees |
| #13 | MeSH descriptor: [General Practice] explode all trees |
| #14 | MeSH descriptor: [General Practitioners] explode all trees |
| #15 | MeSH descriptor: [Physicians, Family] explode all trees |
| #16 | MeSH descriptor: [Physicians] this term only |
| #17 | MeSH descriptor: [Physicians, Primary Care] explode all trees |
| #18 | MeSH descriptor: [Office Visits] this term only |
| #19 | (ambulatory near/3 (care or setting* or facilit* or ward* or department* or service*)):ti,ab,kw or (((general or family) near/2 (practi* or doctor*)) or physician*):ti,ab,kw or ("primary care" or "primary health care" or "primary healthcare"):ti,ab,kw or ("after hour*" or afterhour* or "out of hour*" or ooh):ti,ab,kw or clinic or clinics or visit or visits:ti,ab,kw (Word variations have been searched) |
| #20 | MeSH descriptor: [Community Health Services] this term only |
| #21 | MeSH descriptor: [Community Health Workers] explode all trees |
| #22 | ((health* or medical) near/2 (center* or centre*)):ti,ab,kw or (community near/3 (service* or worker* or volunteer* or aide* or assistant*)):ti,ab,kw or (health near/2 (service* or worker* or volunteer* or aide* or assistant*)):ti,ab,kw (Word variations have been searched) |
| #23 | #11 or #12 or #13 or #14 or #15 or #16 or #17 or #18 or #19 or #20 or #21 or #22 |
| #24 | MeSH descriptor: [Practice Patterns, Physicians'] explode all trees |
| #25 | MeSH descriptor: [Health Knowledge, Attitudes, Practice] explode all trees |
| #26 | MeSH descriptor: [Attitude of Health Personnel] explode all trees |
| #27 | MeSH descriptor: [Guideline Adherence] explode all trees |
| #28 | (barrier* or obstacle* or challeng*):ti,ab,kw or (facilitat* or enabl* or opportunit* or influence* or motivat*):ti,ab,kw or (implement* or uptake or utilis* or utiliz* or adopt*):ti,ab,kw or (attitud* or perception* or perspective* or view* or opinion* or expectatio*? or fear*):ti,ab,kw or (knowledge or understand* or comprehen* or aware* or communicat*):ti,ab,kw (Word variations have been searched) |
| #29 | #24 or #25 or #26 or #27 or #28 |
| #30 | #5 and #10 and #23 and #29 |
