# Supplementary material for: Factors influencing the implementation of cardiovascular risk scoring in primary care: a mixed-method systematic review
Source: Implement Sci. 2020 Jul 20;15:57. doi: 10.1186/s13012-020-01022-x (PMC7370418; doi:10.1186/s13012-020-01022-x)
Supplement: Supplementary file 3 — Additional File 3: Table S2. Characteristics of Included studies | Table S3. Characteristics of Excluded studies and Reasons for exclusion [file 13012_2020_1022_MOESM3_ESM.docx]

**Table S2. Characteristics of Included Studies**

| **_Author and year_** | **_Country_** | **_GNI Classification_** | **_Study design_** | **_Participant characteristics_** | **_Type of cardiovascular assessment tool_** | **_Outcomes measured_** |
| --- | --- | --- | --- | --- | --- | --- |
| **_Quantitative studies_** | | | | | | |
| _Abaci et al. 2010_ | _Turkey_ | _Upper Middle Income_ | _Cross-sectional study_ | _Doctors_ | _ESC SCORE, Framingham, Modified Framingham, ESC Hypertension_ | _Characteristics of the patients and distribution of risk factors, distribution of the guidelines used to manage the cardiovascular risk, reasons for not using any guidelines, use of scoring methods for the calculation of cardiovascular risk and reasons for not using scoring methods._ |
| _Bonnevie et al. 2005_ | _Denmark_ | _High Income_ | _Survey_ | _General Practitioners_ | _Computerised program (PRECARD) that uses the Copenhagen Risk Score_ | _Characteristics of PRECARD users or non-users, how often the program was used with different kinds of patients, the effect of the program on the patients according to general practitioners, reasons for no longer using the program in non-users._ |
| _Byrne et al. 2015_ | _Ireland_ | _High Income_ | _Cross-sectional study_ | _General Practitioners_ | _SCORE system_ | _Demographic characteristics, awareness and use of risk assessment tools and CVD prevention guidelines among Irish GP cohort, barriers to the use of RA tools, barriers to the implementation of CVD prevention guidelines, barriers to optimal management of CVD risk_ |
| _Eichler et al. 2007_ | _Switzerland_ | _High Income_ | _Survey_ | _General Practitioners_ | _PROCAM-score, EU-SCORE, Framingham score, AGLA score (PROCAM-derived Swiss risk score), New Zealand Guidelines_ | _Frequency of specific obstacles for application of cardiovascular prediction rules among physicians_ |
| _Dallongeville et al. 2012_ | _Austria, Belgium, France, Germany, Greece, Norway, Russia, Spain, Sweden, Switzerland, Turkey and the UK_ | _High Income_ | _Survey_ | _Physicians_ | _SCORE system, risk assessment tables contained in the ESC/ESH Guidelines for the Management of Arterial Hypertension, risk assessment systems based on the Framingham study, locally calibrated Framingham study based equations_ | _Characteristics of participating physicians, reasons for not using clinical guidelines among non-users, proportion of physicians using clinical guidelines for CVD prevention, use of global CVD risk factor assessment tools, proportion of physicians using global CVD risk assessment tools, limitation of risk assessment tools, communication strategies usually used for the management of behavioural risk factors and barriers to CVD prevention_ |
| _Elisaf et al. 2014_ | _Greece_ | _High Income_ | _Cross-sectional study_ | _Physicians_ | _ESC SCORE, Framingham, ESC & ESH Hypertension 2007_ | _Characteristics of the physicians, patients and distribution of risk factors, distribution of the guidelines used to manage the cardiovascular risk, reasons for not using any guidelines, use of scoring methods for the calculation of cardiovascular risks,  reasons for not using scoring methods, limitations of risk assessment tools, barriers to CVD primary prevention, achievement of goals among patients treated for the main risk factors in Greece, residual cardiovascular risk in patients achieving treatment goals._ |
| _Elustondo et al. 2013_ | _Spain_ | _High Income_ | _Descriptive transversal study/cross-sectional study_ | _Doctors_ | _Tables; Framingham, ATP III, REGICOR, SCORE_ | _Demographic data, professional circumstances (number of years working in primary care, postgraduate titles, workload), doctors’ opinion on the cardiovascular risk tables, the use of these tables in daily clinical practice and main barriers when using them._ |
| _Imms et al. 2010_ | _Australia_ | _High Income_ | _Survey_ | _General Practitioners_ | _New Zealand Absolute Risk Calculator, Absolute Risk, Joint British Coronary Risk Prediction Chart 2_ | _General practitioners’ demographics, knowledge, cardiovascular risk calculator use, cardiovascular risk factor importance, instrument validation_ |
| _Schmieder et al. 2012_ | _Germany_ | _High Income_ | _Comparative cross-sectional study_ | _Physicians_ | _ESC CVD prevention, ESC/ESH hypertension, ATP III, JNC VII, Local - ESC score, ESC/ESH hypertension, Framingham study, Framingham localised*_ | _Physicians’ attitudes toward risk factor control, patient characteristics and degree of risk factor control, perspectives to improve care_ |
| _Shillinglaw et al. 2012_ | _U.S.A_ | _High Income_ | _Cross-sectional web-based survey_ | _Physicians_ | _Paper charts, web-based, non-web based computer program, program on a personal assistant_ | _Demographics, awareness and use of tools to calculate global CHD risk, physicians who use global CHD risk assessment, reasons for not using global CHD risk assessment._ |
| _Sposito et al. 2009_ | _Brazil, U.S.A, Greece, Chile, Venezuela, Portugal, The Netherlands, Central America (Costa Rica, Panama, El Salvador and Guatemala)_ | _Upper Middle Income, High, Lower Middle Income_ | _Cross-sectional study_ | _Physicians_ | _FRS, Heart Score, PROCAM, JBS score_ | _Respondent characteristics, physicians’ presumption of CVD risk, regular use of CVD risk scores, reasons for non-use of CVD risk scores, indication for lipid-lowering therapy,_ |
| _Tawfik et al. 2015_ | _Egypt_ | _Lower Middle Income_ | _Cross-sectional study_ | _Physicians_ | _The WHO CVR score, Framingham, British risk score._ | _Demographics, awareness and use of the cardiovascular risk scoring tools, type of tools used, barriers to use, cardiovascular risk factors._ |
| **_Qualitative studies_** | | | | | | |
| ^Bonner et al 2013^ | ^Australia^ | ^High Income^ | ^Qualitative; semi-structured interviews; framework analysis^ | ^General Practitioners^ | ^Unclear^ | ^Use of different cardiovascular risk assessment strategies^ |
| ^Doolan-Noble et al 2010^ | ^New Zealand^ | ^High income^ | ^Qualitative; focus group discussions; thematic analysis^ | ^GPs and Practice Nurses^ | ^Predict, EDGE^ | ^The patient, primary health care providers, the general practice, the health care system^ |
| ^Liew et al 2013^ | ^United Kingdom^ | ^High Income^ | ^Qualitative; face to face interviews; thematic analysis^ | ^General Practitioners^ | ^Framingham, QRISK^ | ^What do practitioners say the risk scores predict? Do practitioners use risk scores in patients already on treatment?  How do practitioners take account of treatment? How do practitioners choose which score to use? Do practitioners think score accuracy matters?^ |
| ^Torley et al. 2005^ | ^Australia^ | ^High Income^ | ^Qualitative; focus group discussions; thematic analysis^ | ^General Practitioners^ | ^New Zealand risk score, Medical Director, Joint British Coronary Risk Prediction Charts.^ | ^Respondent demographics, current methods of cardiovascular risk assessment, current use risk of assessment tools, problems with cardiovascular risk assessment tools, absolute vs relative risk^ |
| ^Vaidya et al. 2004^ | ^Australia^ | ^High Income^ | ^Qualitative, semi-structured interviews; thematic analysis^ | ^General Practitioners, patients^ | ^Absolute cardiovascular risk assessment - unclear about the exact tools^ | ^Risk communication, factors promoting/preventing change over 12 months in patients and physicians, factors influencing the maintenance of use in General Practitioners, impact on management after 12 months^ |
| ^Van Steenkiste et al. 2004^ | ^The Netherlands^ | ^High Income^ | ^Qualitative; semi-structured in-depth interviews; thematic analysis^ | ^General Practitioners^ | ^Risk tables^ | ^Demographics, barriers relating to the guideline-Risk table as an instrument, barriers relating to the guideline/content of the risk table (recommendations), attitude, routines, knowledge, skills, society, medical profession, practice organisation.^ |
| ^Van Steenkiste et al. 2004^ | ^The Netherlands^ | ^High Income^ | ^Qualitative; semi-structured in-depth interviews; thematic analysis^ | ^Patients^ | ^Risk tables^ | ^Demographics, barriers related to patients’ ideas about cardiovascular disease and risk factors, barriers related to patients’ risk perception (fears), expectations for information and treatment^ |
| ^Wan et al 2008^ | ^Australia^ | ^High Income^ | ^Qualitative; focus group discussions and interviews; thematic analysis^ | ^GPs, patients and key informants^ | ^New Zealand CVAR electronic and paper-based calculators^ | ^Demographic information, conducting a cardiovascular risk assessment in a General Practitioners’ consultation, patient self-assessment, shared approach in CVAR assessment and management^ |
| ^Wan et al 2008^ | ^Australia^ | ^High Income^ | ^Qualitative; focus group discussions; thematic analysis^ | ^GPs and patients^ | ^New Zealand CVAR electronic and paper-based calculators^ | ^Demographics, patient age, risk factors and wellbeing, pre-existing GP-patient relationship, patient awareness and motivation, initiation of consultation for CVAR assessment^ |
| _Mixed methods studies_ | | | | | | |
| _Collins et al. 2017_ | _Jordan_ | _Lower Middle Income_ | _Mixed methods; quantitative (descriptive) and qualitative (one on one, face to face, semi-structured interviews; thematic analysis) strands of equal priority, integrated during data collection and interpretation_ | _Doctors, nurses, health promoters, pharmacists, and managers_ | _WHO/ISH CVD risk chart_ | _Quantitative: the WHO/ISH CVD risk distribution of the included population, lipid-lowering treatment prescribing patterns based on calculated CVD risk category, agreement between documented and calculated WHO/ISH CVD risk scores and agreement between documented and calculated WHO/ISH CVD after aggregating by the clinically significant threshold of WHO/ISH risk of 20%, where individuals with a history of CVD are categorised as high risk._  _Qualitative: summary of qualitative findings with example quotations._ |
| _Ferrante et al. 2013_ | _Argentina_ | _Upper Middle Income_ | _Mixed methods; qualitative (in-depth interviews following a grounded theory framework) and a cross-sectional before and after analysis study_ | _Physicians, nurses, social workers, primary health care area coordinators/managers, county secretaries of health_ | _WHO/ISH_ | _Barriers to preventing cardiovascular disease and implementing clinical practice guidelines in primary care, the impact of implementing a tailored enactment of the adapted WHO guidelines to prevent cardiovascular diseases._ |
| _Kirby et al. 2009_ | _United Kingdom_ | _High Income_ | _Mixed methods; survey and qualitative study (focus group discussions and semi-structured interviews; thematic analysis)_ | _GPs, practice nurses, nurse practitioners and patients_ | _JBS 2 paper charts and calculator_ | _Use of Joint British Societies 2 paper risk charts and electronic cardiovascular risk assessment calculator, reasons for discontinuing use of the electronic calculator and factors that would have facilitated the use of it, patients' understanding of the use of cardiovascular risk tools._ |
| _Oriol-Zerbe et al. 2007_ | _Germany_ | _High Income_ | _Mixed methods; quantitative (descriptive cross-sectional study) and qualitative (semi-structured interviews; thematic analysis)_ | _Physicians_ | _US guideline (NCEP III) – Framingham Risk Score, European guideline for risk scoring_ | _Calculation of coronary risk, Summary of comparison between guidelines/risk, use of risk-calculator charts and guidelines for primary prevention, Barriers for prescribing lipid-lowering therapy in primary prevention, tabulations and prescribing by GPs, In what case was the diagnosis of hyperlipidemia given to the patient? Reasons for prescribing/not prescribing._ |

**Table S3. Characteristics of Excluded studies and Reasons for exclusion**

| **Author and year** | **Country** | **Title** | **Reason for exclusion** |
| --- | --- | --- | --- |
| Adang et al., 2016 | Netherlands | *Efficiency of the implementation of cardiovascular risk management in primary care practices: an observational study* | Wrong intervention |
| Ahmed et al., 2013 | Saudi Arabia | *Evaluation of risk factors for cardiovascular diseases among Saudi diabetics’ patients attending primary health care service* | Wrong outcomes |
| Al-Mohtaseb et al., 2011 | - | *What do general practitioners know about total cardiovascular risk assessment tool SCORE European Heart Journal* | The full paper was not available |
| Banegas et al., 2011 | Spain | *Physicians attitudes and frequency of traditional cardiovascular risk factors in primary prevention in Spain: Spanish results of the EURIKA study* | The full paper was not available |
| Bonner et al., 2014 | Australia | *I don't believe it, but I'd better do something about it: Patient experiences of online heart age risk calculators* | Wrong intervention |
| Brotons et al., 2013 | Spain | *Implementation of Spanish adaptation of the European guidelines on cardiovascular disease prevention in primary care* | Wrong intervention |
| Bucki et al., 2014 | Australia | *Finding enablers and knowing barriers - Facilitating primary health care nurses to implement an absolute CVD risk approach in general practice* | The full paper was not available |
| Byrne et al., 2012 | Ireland | *Preventing cardiovascular disease (CVD): A survey of Irish general practitioners’ awareness of risk assessment tools and cardiovascular disease prevention guidelines* | The full paper was not available/Duplicate to Byrne et al. 2015 on reading abstract |
| Dallongeville et al., 2010 | - | *A survey of physicians' attitudes toward the control of cardiovascular risk factors. The eurika study* | The full paper was not available |
| Dallongeville et al., 2010 | France | *Physicians’ attitudes and frequency of traditional cardiovascular risk factors in primary prevention in France. The EURIKA study* | The full paper was not available |
| Delpech et al., 2016 | France | *Primary prevention of cardiovascular disease: More patient gender-based differences in risk evaluation among male general practitioners* | Wrong outcomes |
| Du et al., 2010 | - | *Identifying barriers toward implementing absolute cardiovascular risk assessment tools in primary care setting* | The full paper was not available |
| George et al., 2016 | India | *Barriers to cardiovascular disease risk reduction: Does physicians' perspective matter?* | Wrong outcomes |
| Graham et al., 2006 | Germany, France, Italy, Spain, the UK and Poland | *Factors impeding the implementation of cardiovascular prevention guidelines: findings from a survey conducted by the European Society of Cardiology* | Wrong intervention |
| Gupta et al, 2009 | Australia | *Cardiovascular risk assessment in Australian general practice* | Wrong outcomes |
| Hobbs et al., 2010 | Europe | *Barriers to cardiovascular disease risk scoring and primary prevention in Europe* | Wrong study design |
| Khambatta et al., 2010 | U.S. A | *Recognition of cardiovascular risk factors and implementation of primary prevention interventions with utilization of an on-line form* | The full paper was not available |
| Muylder et al., 2004 | Belgium | *Obstacles to cardiovascular prevention in general practice* | Wrong outcomes |
| Manfrini et al., 2007 | Italy | *Barriers to clinical risk scores adoption* | Wrong study design |
| Matangi et al., 2013 | Canada | *Awareness and use of cardiovascular risk scores by family physicians in south-eastern Ontario* | Wrong outcomes |
| Matangi et al., 2013 | Canada | *Knowledge and implementation of cardiovascular risk scores by family physicians* | The full paper was not available |
| McKillop et al., 2010 | New Zealand | *Barriers and Enablers to implementation of a New Zealand-wide guideline for assessment and management of cardiovascular risk in primary health care: A template analysis* | Wrong intervention |
| Mendis et al., 2004 | Nigeria | *Barriers to management of cardiovascular risk in a low-resource setting using hypertension as an entry point* | Wrong outcomes |
| Miserez et al., 2011 | Switzerland | *A survey of physician's attitudes towards the control of cardiovascular risk factors. Swiss results of the EURIKA study* | The full paper was not available |
| Mohtaseb et al., 2013 | - | *Total cardiovascular risk assessment: What do doctors know about?* | The full paper was not available |
| Murray et al., 2013 | Canada | *Knowledge and implementation of cardiovascular risk scores by family physicians in southeastern Ontario* | The full paper was not available |
