# Supplementary material for: Factors influencing the implementation of cardiovascular risk scoring in primary care: a mixed-method systematic review
Source: Implement Sci. 2020 Jul 20;15:57. doi: 10.1186/s13012-020-01022-x (PMC7370418; doi:10.1186/s13012-020-01022-x)
Supplement: Supplementary file 4 — Additional File 4: Figure S1. PRISMA Flowchart [file 13012_2020_1022_MOESM4_ESM.docx]

**Figure S1**. PRISMA Flowchart

Identification

Additional records identified through other sources
(*n* = 0)

Records identified through database searching
(*n* = 4659)

Records screened for Title and Abstract

(*n*=3010)

Included

Screening

Eligibility

Studies included for synthesis
(*n* = 25)

Full-text articles assessed for eligibility
(*n* = 52)

Full-text articles excluded, with reasons
(*n* = 27)

*10 - Could not access full text*

*Six - Wrong outcomes*

*Three - Duplicate*

*Five - Wrong intervention*

*Two - Wrong study design*

*One was a QI project that was made an abstract but no paper was produced.*

*One - Wrong patient population*

Records excluded
(*n* = 2958)

Records after duplicates removed
(*n* = 3010)
