# Supplementary material for: Factors influencing the implementation of cardiovascular risk scoring in primary care: a mixed-method systematic review
Source: Implement Sci. 2020 Jul 20;15:57. doi: 10.1186/s13012-020-01022-x (PMC7370418; doi:10.1186/s13012-020-01022-x)
Supplement: Supplementary file 5 — Additional File 5: Table S4. Summary of Facilitators and Barriers as coded on the CFIR [file 13012_2020_1022_MOESM5_ESM.docx]

**Table S4**. Summary of Facilitators and Barriers as coded on the CFIR

| CFIR Domains (definition and constructs) | Facilitators | Barriers |
| --- | --- | --- |
| **Intervention characteristics** - *Key attributes of an intervention*  Intervention source, evidence strength & quality, relative advantage, adaptability, trialability, complexity, design quality & packaging and cost | - Based on the latest scientific evidence - (C Bonner *et al.*, 2013; Elustondo *et al.*, 2013; S M Liew *et al.*, 2013)   ‘I kind of assumed that, maybe naively, that the data is updated ... I wouldn’t pretend to understand … I think intuitively it makes sense that the older data is probably going to be more useful but I don’t know.’ – GP (S M Liew *et al.*, 2013)   - Perceived to predict CVD risk accurately and include the main risk factors for cardiovascular disease – (Elustondo *et al.*, 2013) - Evidence that the tools better therapeutic decisions - (Wan *et al.*, 2008a; M Kirby and Machen, 2009; Dallongeville *et al.*, 2010; Shillinglaw, Anthony J Viera, *et al.*, 2012; Carissa Bonner *et al.*, 2013; Elustondo *et al.*, 2013; D Byrne *et al.*, 2015; Collins *et al.*, 2017)   ‘It depends on the level of risk. If the risk is rela-  tively low you would give advice that’s relevant to that aspect of risk, and if the risk is high you then you obviously have to think of what things to put in place immediately, down the track, follow up (GP group 1).’’ - (Wan *et al.*, 2008b)   - Evidence that the tools improve patient care - (Wan *et al.*, 2009; Shillinglaw, Anthony J. Viera, *et al.*, 2012)   ‘I think the strength of the absolute risk concept  is that it improves the targeting of certain interventions, so that you have a greater accuracy when you’re prescribing things like Statins but also a greater accuracy and conﬁdence when you prescribe just behavioural measures like diet and exercise… (KI 6).’’ - (Wan *et al.*, 2008b)   - Evidence that the tools reduce CVD risk adequately - (M. Kirby and Machen, 2009) - Perceived as beneficial - (Shillinglaw, Anthony J. Viera, *et al.*, 2012; Collins *et al.*, 2017)   “I think I’m using it with most of patients because the score gives you a clear idea about the risk and how to react so I depend on it a lot.”– Doctor B (Collins *et al.*, 2017)   - Helps understand CVD risk - (Bonnevie, Thomsen and Jorgensen, 2005; Oriol-Zerbe and Abholz, 2007; M. Kirby and Machen, 2009; Vaidya *et al.*, 2012; Elustondo *et al.*, 2013; Su May Liew *et al.*, 2013; D Byrne *et al.*, 2015; Collins *et al.*, 2017)   “So we are just, I mean showing indication that you are in the green area, [...] so you don’t need to take aspirin because you might have side effects more than the benefits from aspirin and actually a lot of them they are convinced [...]. So it is very helpful and it is convincing.”– Doctor E (Collins *et al.*, 2017)   - Helps to motivate patients - (Bonnevie, Thomsen and Jorgensen, 2005; Vaidya *et al.*, 2012; Carissa Bonner *et al.*, 2013; D Byrne *et al.*, 2015)   “I actually do use the risk calculator to give them a percentage . . . I use the calculator for, as a bit of grounding for me but also as a way of motivating a patient” (ID16, male, 9 years’ experience) - (Carissa Bonner *et al.*, 2013)   - Helps improve follow up - (Bonnevie, Thomsen and Jorgensen, 2005) - Helps in educating patients - (Torley *et al.*, 2005; Dallongeville *et al.*, 2010)   ...I’ve got one program where you can  show the patient how the risk changes as you run the blood pressure down, or change the cholesterol. It’s quite a powerful tool...’ - (Torley *et al.*, 2005)   - Tools are a checklist to avoid missing something - (Oriol-Zerbe and Abholz, 2007; Vaidya *et al.*, 2012; Carissa Bonner *et al.*, 2013)   Before, I probably would not have instituted treatment, because their blood pressure wasn’t that high and their lipids weren’t that bad. But now when you see the risk score, it has probably made you think I’d better start treatment. – GP - (Vaidya *et al.*, 2012)   - Tools increased compliance to advice given to patients in consultations - (M. Kirby and Machen, 2009) - The use of the tools was perceived as feasible in daily practice - (Elustondo *et al.*, 2013) - The tools are easy to use - (M. Kirby and Machen, 2009; Vaidya *et al.*, 2012; Su May Liew *et al.*, 2013)   It is not difficult to use, doesn’t take a long time. They can see it in front of them. They like it even more because it is on the computer. – GPs - (Vaidya *et al.*, 2012)   - Tools are easily understood by patients - (Vaidya *et al.*, 2012)   You know, to me 11, 12 percent. I would assume that anybody with no heart problems would have that sort of risk. If it had gone into the 20s and 30s I would have been worried. – patient - (Vaidya *et al.*, 2012)   - The use of charts - (Dallongeville *et al.*, 2010; Shillinglaw, Anthony J. Viera, *et al.*, 2012; C Bonner *et al.*, 2013)   “I think the doctors who don’t use paper-based things, they are quite used to [information technology], they are using [absolute risk], but for us like I am in the middle-aged group, still using paper work so it’s a bit of an effort” (passive disregard) - (C Bonner *et al.*, 2013)   - Use of colours to classify risk - (Collins *et al.*, 2017)   “From my experience the colours are best for our  patient from numbers.” – Nurse E - (Collins *et al.*, 2017)   - The use of software - (Dallongeville *et al.*, 2010) - Using a web based application - (Shillinglaw, Anthony J. Viera, *et al.*, 2012) - Use of a program on a PDA or smartphone - (Torley *et al.*, 2005; Shillinglaw, Anthony J. Viera, *et al.*, 2012) - Having a computer prompt - (M. Kirby and Machen, 2009) - Incorporating the tool into the GPs clinical systems - (M. Kirby and Machen, 2009; Vaidya *et al.*, 2012)   We have our own medical program. To use something else on top of that is quite cumbersome. It’s gotta be incorporated otherwise it is very unlikely I will ever use it. – GP - (Vaidya *et al.*, 2012) | - Distrust of their predictive ability -(Eichler *et al.*, 2007; Elustondo *et al.*, 2013; Tawfik *et al.*, 2015)   GP8: ‘So you take your population at year zero, you follow them all through but you don’t actually know who’s been treated. Oh gosh, yes, so that actually brings huge inaccuracy into the risk scores then.’ - (Su May Liew *et al.*, 2013)   - Perceived interference by other stakeholders - (Tawfik *et al.*, 2015) - Lack of consensus on the use of the tools - (Doolan-Noble, Mann and Tracey, 2010; Elustondo *et al.*, 2013)   “I’ve seen three changes in long-term condition strategies, at a government level. And there is no consistency going down the line about how we should work within those strategies…” (F2) - (Doolan-Noble, Mann and Tracey, 2010)   - Perceived lack of prediction accuracy resulting to overestimation or underestimation- (Eichler *et al.*, 2007; Abaci, 2010; Dallongeville *et al.*, 2010; Su May Liew *et al.*, 2013; Elisaf *et al.*, 2014)   GP3: ‘It’s obvious when you think about it, that there’s an issue about how treatment is going to change outcomes. And that’ll interfere with prediction.’ - (Su May Liew *et al.*, 2013)   - Tools do not consider some important risk factors - (Abaci, 2010; Dallongeville *et al.*, 2010; Carissa Bonner *et al.*, 2013; Elisaf *et al.*, 2014; Tawfik *et al.*, 2015)   ‘The calculator of course doesn’t include certain factors . . . if someone does do a lot of exercise I would . . . think their risk is probably lower’ (ID16, male, 9 years’ experience) - (Carissa Bonner *et al.*, 2013)   - Tools focus too much on risk - (Bonnevie, Thomsen and Jorgensen, 2005) - Tools do not consider the complexity of the patient - (Bonnevie, Thomsen and Jorgensen, 2005; Eichler *et al.*, 2007; Oriol-Zerbe and Abholz, 2007; Tawfik *et al.*, 2015)   ‘We actually know the patient quite well and their background and their habits now so it’s easier for us to make an overall assessment. (ID6,  female, 19  years’ experience)’ - (Carissa Bonner *et al.*, 2013)   - Tools lead to over treatment or under treatment - (Eichler *et al.*, 2007; Elustondo *et al.*, 2013; Tawfik *et al.*, 2015) - Tools are rapidly outdated - (Ben van Steenkiste *et al.*, 2004; Oriol-Zerbe and Abholz, 2007; Ferrante *et al.*, 2013)   “At this health center we don’t have  enough time to keep us up to date with new evidence of guidelines.” [attending family physician] - (Ferrante *et al.*, 2013)   - Tools are perceived to not have any beneficial use - (Abaci, 2010; Dallongeville *et al.*, 2010; Roland E Schmieder, Goebel and Bramlage, 2012; Shillinglaw, Anthony J. Viera, *et al.*, 2012; Elisaf *et al.*, 2014) - Tools are perceived as less superior to clinical judgement - (Oriol-Zerbe and Abholz, 2007; A Imms, Quinn and Nelson, 2010; C Bonner *et al.*, 2013; Elustondo *et al.*, 2013)   [AR assessment] doesn’t take into account your family history, your weight, if you’re active or not . . . when you’ve been in this game for as many years as I have you like to get a big picture.  (ID22, male, 22 years’ experience) - (C Bonner *et al.*, 2013)   - Tools are time consuming - (Eichler *et al.*, 2007; M Kirby and Machen, 2009; Sposito *et al.*, 2009a; Abaci, 2010; Dallongeville *et al.*, 2010; Roland E Schmieder, Goebel and Bramlage, 2012; Shillinglaw, Anthony J. Viera, *et al.*, 2012; Elustondo *et al.*, 2013; Elisaf *et al.*, 2014; Tawfik *et al.*, 2015) - Tools prolonged consultations - (Bonnevie, Thomsen and Jorgensen, 2005; Collins *et al.*, 2017)   ‘’So if it’s the first time the patient is coming I need to wait for the labs to calculate what the percentage is.” – Doctor A - (Collins *et al.*, 2017)   - Tools lowered the quality of the consultation - (Bonnevie, Thomsen and Jorgensen, 2005) - Tools do not allow the calculation of risk in the elderly - (Abaci, 2010; Dallongeville *et al.*, 2010; Elisaf *et al.*, 2014) - The risk duration calculated by the tools are too long - (Abaci, 2010; Elisaf *et al.*, 2014) - Tools interfere with the GPs decision making process - (Bonnevie, Thomsen and Jorgensen, 2005) - Some of the risk factors required for risk prediction are not measured in the practice - (Eichler *et al.*, 2007; Ferrante *et al.*, 2013)   “At our primary health care centres, we do not have appropriate resources to follow cardiovascular guidelines, particularly with keeping an information system and performing laboratory tests.” [nurse] - (Ferrante *et al.*, 2013)   - Explaining the results of the tool to patients is complicated – (Van Steenkiste, Van Der Weijden, Stoffers, *et al.*, 2004; Torley *et al.*, 2005; Noble *et al.*, 2013)   The trouble with such a table is that you cannot discuss it with patients. Patients are generally not familiar with statistics, so for nine out of ten of them reading a graph is not something they  Implementing cardiovascular risk tables in general practice are used to. Let alone interpreting tables with red, yellow and white colours. (GP4) - (Van Steenkiste, Van Der Weijden, Stoffers, *et al.*, 2004)   - The prediction rules are unclear - (Eichler *et al.*, 2007; S M Liew *et al.*, 2013)   GP18: ‘If someone’s on blood pressure treatment, do you say that treatment of blood pressure reduces [the assessed risk] by two thirds and therefore you need to modify for the third as not being attenuated? It’s all very, very grey, isn’t it?’ - (S M Liew *et al.*, 2013)   - The tool has technical problems - (Bonnevie, Thomsen and Jorgensen, 2005) - The tool does not communicate with other programs - (Bonnevie, Thomsen and Jorgensen, 2005) |
| **Outer setting** – *includes;*  Patients’ needs & resources, cosmopolitanism, peer pressure and external policy & incentives | Patients’ demand for risk assessment - (Van Steenkiste, Van Der Weijden, Stoffers, *et al.*, 2004; Torley *et al.*, 2005; Wan *et al.*, 2010)  ‘…But if I decide not to test them, it just means you spend 10 minutes explaining why it’s not necessary. Three months later they turn up at your office and say they want it anyway. That annoys me, especially since I’ve spent an extra 10 minutes on them. I’ve given up fighting it. (GP8)’ - (B van Steenkiste *et al.*, 2004)  If I want to know, I feel it’s my right to know - (Van Steenkiste, Van Der Weijden, Timmermans, *et al.*, 2004a)   - Patients’ perception of cardiovascular risk assessment as essential - (Wan *et al.*, 2010)   ‘A media campaign would make it easier for us, because to start with some of the patients may consider this is just a way of us getting more business out of them, and that would make it not valuable for them, but when they come and ask for it because they’ve seen it in the media they make a lot of value and they will be more motivated and more committed... (GP FG3) ‘ -(Wan *et al.*, 2010)   - Patients’ relationship with the GP - (Wan *et al.*, 2010)   ‘If they’re just a ﬂoating patient coming to see you for a cough or cold or whatever it is, they probably have a regular doctor to go back to and follow through, but if you have some problems with your regular doctor, I’ll ﬁnd some time to go through it with you at least then you can get their conﬁdence and show them a reason...it depends how you approach them. (GP FG1)’ - (Wan *et al.*, 2010)   - The feeling that the society has the right through approved guidelines and tools - (Oriol-Zerbe and Abholz, 2007) - The focus of funding by external organisations – (Doolan-Noble, Mann and Tracey, 2010)   “…if I am blunt about it, guided by where we get our funding from. Care Plus gives us funding. Diabetic Project gives us funding. Immunisation gives us funding.” (F3) - (Doolan-Noble, Mann and Tracey, 2010)   - Available supportive care pathways/programs and their longevity - (Doolan-Noble, Mann and Tracey, 2010)   “There’s a really good pathway that was set up when, before we started this, which was, you know, the free GP visit… the dietician visit, the Green Prescription. And it’s really good.” (F2) - (Doolan-Noble, Mann and Tracey, 2010)  “Short-term contracts to run pilots, which just as you are gaining some traction the contract runs out, funding ceases and the programme falls over.” (F1) - (Doolan-Noble, Mann and Tracey, 2010)   - Patients’ access to information - (Wan *et al.*, 2010)   ‘It’s a lot easier to look after your health now, because they have all these programs, there is the... Program, walking groups, all these places where you can get education. (Patient FG3) - (Wan *et al.*, 2010) | - Patients focus on a single risk factor not the total risk - (D Byrne *et al.*, 2015) - Patients do not want to know their cardiovascular risk - (Van Steenkiste, Van Der Weijden, Timmermans, *et al.*, 2004b; Eichler *et al.*, 2007; Tawfik *et al.*, 2015; Collins *et al.*, 2017)   “We tell them that I like to add statin a new medication that’s called statin, it’s for cholesterol. And they usually react”, “I don’t have any cholesterol and no I don’t have any problems with my cholesterol,” or “something like that.”– Doctor A - (Collins *et al.*, 2017)  I think that is wrong; you go there for some other disease and they still challenge you about your smoking. If I’m told to stop smoking they should also provide me with the means to quit. – patient - (Van Steenkiste, Van Der Weijden, Timmermans, *et al.*, 2004b)   - Patients may not afford some of the investigations required by the tool - (Tawfik *et al.*, 2015) - Patients’ personal circumstances and experiences - (Doolan-Noble, Mann and Tracey, 2010; Vaidya *et al.*, 2012; Collins *et al.*, 2017)   “Some patients gradually decrease their cigarettes, but suddenly when they come”“I increased my consumption.”“Why?”“Because my brother left away in Syria, because my son died, because I need more money.”– Nurse B - (Collins *et al.*, 2017)   - Patients health priorities as perceived by them or by the clinicians - (Wan *et al.*, 2008b; Doolan-Noble, Mann and Tracey, 2010; Ferrante *et al.*, 2013)   “Patients usually prioritize urgent health problems and not prevention actions. We have to respond to this demand.” [decision maker] - (Ferrante *et al.*, 2013)  “So as a doctor my first priority is actually to treat the illness… and when I’m treating illness, my first priority is to treat the most urgent illness first.” (F3) - (Doolan-Noble, Mann and Tracey, 2010)   - Patients’ perceptions and understanding of cardiovascular risk - (B van Steenkiste *et al.*, 2004; Wan *et al.*, 2008b; Carissa Bonner *et al.*, 2013)   ‘As a result, the general public have unrealistic ideas about the contribution of cholesterol to the absolute cardiovascular risk, and the topic is fraught with anxiety. This whole cholesterol story is based on people’s anxiety. If you’re anxious, get your cholesterol level tested. It used to be: get your blood pressure tested. It’s all to do with anxiety. It is linked to something it shouldn’t be linked to. (GP5) - (Van Steenkiste, Van Der Weijden, Stoffers, *et al.*, 2004)   - Patients demand for treatment regardless of cardiovascular risk - (Ben van Steenkiste *et al.*, 2004; Collins *et al.*, 2017)   ‘If my blood pressure is too high, I should be given medication, whether I’m in the high-risk group or not.’ - (Ben van Steenkiste *et al.*, 2004)  “Yes. Many of our women didn’t want to follow diet regimen or DM diet… they want medication to lose weight and they ask about medication.” // “I told them that it’s not useful…but many of them search about them and bring and use.”– Nurse E - (Collins *et al.*, 2017)   - Patients’ environment and surroundings - (Doolan-Noble, Mann and Tracey, 2010; Collins *et al.*, 2017)   “The environment many of our patients live in is not conducive to making lifestyle behavioural changes… multiple fast food outlets, pavements may not be safe, lack of cycle ways etc.” (F1) - (Doolan-Noble, Mann and Tracey, 2010)   - Patients’ perceptions and understanding of cardiovascular disease and treatment - (Van Steenkiste, Van Der Weijden, Timmermans, *et al.*, 2004c; Wan *et al.*, 2008a; Collins *et al.*, 2017)   ““If I drink a cup of water plus one tablespoon of vinegar, it’s bad on the cholesterol?” This is a common question.” // “It’s something usually in Facebook.”– Person A - (Collins *et al.*, 2017)  “Sometimes they accept the neighbours’ opinion more than us.”– Nurse A   - The use of these tools may be misused externally by health authorities (Tawfik *et al.*, 2015) - There are no adequately structured systems in the clinic to ensure primary prevention in CVD i.e. lack of staff, inadequate budgeting, lack of interest by managers and inadequate encouragement of doctors - (Abaci, 2010; Dallongeville *et al.*, 2010; Doolan-Noble, Mann and Tracey, 2010)   “Ministry talks a lot about prevention of illness and disease, but they just don’t fund it. It’s expected to happen.” (F2) - (Doolan-Noble, Mann and Tracey, 2010)   - Health system’s priorities - (Doolan-Noble, Mann and Tracey, 2010; Ferrante *et al.*, 2013; Collins *et al.*, 2017)   “We are overwhelmed with urgent social and health problems and we can’t prioritize prevention activities.” [social worker] - (Ferrante *et al.*, 2013)  “Our model of care is based on satisfying the demand of patients, mainly acute problems, without a focus on chronic condition follow up.” [decision maker] - (Ferrante *et al.*, 2013)   - Conflicting priorities between stakeholders - (B van Steenkiste *et al.*, 2004)   ‘’If we’d really do primary prevention, you’d have to follow hundreds of people for maybe ten or thirty years to prevent one event. And you don’t know who it’s going to be, which is a big problem because it results in lots of tension and disappointment. Let the local health authorities take care of that, that’s fine with me.’’ (GP10) - (B van Steenkiste *et al.*, 2004) |
| **Inner setting** - *The inner setting is recognized as an active interacting facet which includes;*  Structural characteristics, networks and communications, culture, implementation climate and readiness for implementation | - A sense of group identification with the risk and disease for patients - (Collins *et al.*, 2017)   ““I think the refugees become more relax when they talk together, see same cases like”, “I’m not the only one. I have cardio or I have DM there’s a lot of person like me.” It’s more unique. I think it’s more effective than the individual session.”– Person A - (Collins *et al.*, 2017)   - Organisation the prevention activities to which patients are referred - (Doolan-Noble, Mann and Tracey, 2010)   “…if I sent them to a dietician, or to Pacific Health for Quitline or Smokefree they don’t go, but if it’s here somehow I think it’d be easier.” (F3) - (Doolan-Noble, Mann and Tracey, 2010)   - The availability of the tools during consultation - (C Bonner *et al.*, 2013)   I could just never find them [AR charts] when I needed them so . . . it’s being able to put your  finger on it when you need it and if it was available. I don’t even know if it’s available on our computer program. (ID1, female, 16 years’ experience) - (C Bonner *et al.*, 2013) | - Lack of time to assess for risk associated with high workload - (Wan *et al.*, 2010; Ferrante *et al.*, 2013)   “We have a lot of workload in order to allow us to review guidelines for each visit.” [nurse] - (Ferrante *et al.*, 2013)  I think sometimes they’ve got so many patients that and they’ve got so much to do, and they look at you, and they, you know? They actually don’t test you for some of the things that I think they should... it’s been my initiative for nearly everything that’s wrong...I’ve found that I had to ask for a lot of things. (Patient FG2) - (Wan *et al.*, 2010)   - Lack of close collaboration between health care workers - (Ferrante *et al.*, 2013)   “We usually don’t work in close collaboration with physicians and administrative staff due to communication problems due to our work overload.” [administrative staff] - (Ferrante *et al.*, 2013)   - Lack of health information systems - (Ferrante *et al.*, 2013)   “We don’t have information systems in order to help us to follow up and manage patients with risk factors.” [attending family physician] - (Ferrante *et al.*, 2013)   - Inadequate resources to support risk assessment - (Eichler *et al.*, 2007; Abaci, 2010; Dallongeville *et al.*, 2010; Elustondo *et al.*, 2013; Ferrante *et al.*, 2013; Collins *et al.*, 2017)   “At our primary health care centers, we do not have appropriate resources to follow cardiovascular guidelines, particularly with keeping an information system and performing laboratory tests.” [nurse] - (Ferrante *et al.*, 2013)   - The difficulty of communicating preventive care and self-management to patients by clinicians - (Doolan-Noble, Mann and Tracey, 2010)   “The concept of preventive care and ‘self-management’ is often a difficult one to impart.” (F3) - (Doolan-Noble, Mann and Tracey, 2010)   - Practice culture and professional hierarchy - (B van Steenkiste *et al.*, 2004)   Specialists have greater influence, in the sense that changing the treatment initiated by a specialist is very difficult for a GP. There are lots of people who’ve been to see the cardiologist for an a-typical angina, who didn’t actually have angina, but a transiently abnormal lipid spectrum, and who comes back with a statin. You just try and reverse that. It’s impossible. (GP1) - (B van Steenkiste *et al.*, 2004)   - The patients’ socio-economic status -(Collins *et al.*, 2017)   “Sometimes our patients are shamed to tell you about that. Just you hear”“you must take vegetables, fruit just one time a week.”“They are still silent, because sometimes they do not have anything. That is the problem. It is better, I think, in the home visit to give a good picture or clear picture”– Nurse F - (Collins *et al.*, 2017)   - Lack of knowledge about the tools - (Ferrante *et al.*, 2013)   “I am not aware of any guide to manage risk factors.” [nurse] - (Ferrante *et al.*, 2013) |
| **Characteristics of individuals** - *Organisations are made up of individuals. Setting and intervention constructs are rooted, ultimately, in the actions and behaviours of individuals.* *These include;*  Knowledge & belief about the intervention, knowledge & belief about the disease and risk factors, self-efficacy, individual stage of change, individual identification with organisation and other personal attributes. | - That the intervention was evidence-based - (C Bonner *et al.*, 2013)   I’m comfortable to be guided by the experts rather than try and invent too much on what might be dodgy assumptions on my part. (ID31, male, 30 years’ experience) - (C Bonner *et al.*, 2013)   - Clinician perception and understanding of cardiovascular risk and disease - (Torley *et al.*, 2005; Eichler *et al.*, 2007; A Imms, Quinn and Nelson, 2010; Vaidya *et al.*, 2012; Ferrante *et al.*, 2013; Collins *et al.*, 2017)   ‘Instead of going in hard to the 80 year  olds... we should be going in for longer for the 40 year olds who would not die younger’. - (Torley *et al.*, 2005)  Before, I probably would not have instituted treatment, because their blood pressure wasn’t that high and their lipids weren’t that bad. But now when you see the risk score, it has probably made you think I’d better start treatment. – GP - (Vaidya *et al.*, 2012)   - Helps understand CVD risk - (Bonnevie, Thomsen and Jorgensen, 2005; Oriol-Zerbe and Abholz, 2007; M. Kirby and Machen, 2009; Vaidya *et al.*, 2012; Elustondo *et al.*, 2013; Su May Liew *et al.*, 2013; D Byrne *et al.*, 2015; Collins *et al.*, 2017)   “So we are just, I mean showing indication that you are in the green area, [...] so you don’t need to take aspirin because you might have side effects more than the benefits from aspirin and actually a lot of them they are convinced [...]. So it is very helpful and it is convincing.”– Doctor E (Collins *et al.*, 2017)   - Helps to motivate patients - (Bonnevie, Thomsen and Jorgensen, 2005; Vaidya *et al.*, 2012; Carissa Bonner *et al.*, 2013; D Byrne *et al.*, 2015)   “I actually do use the risk calculator to give them a percentage . . . I use the calculator for, as a bit of grounding for me but also as a way of motivating a patient” (ID16, male, 9 years’ experience) - (Carissa Bonner *et al.*, 2013)   - Helps improve follow up - (Bonnevie, Thomsen and Jorgensen, 2005) - Helps in educating patients - (Torley *et al.*, 2005; Dallongeville *et al.*, 2010)   ...I’ve got one program where you can  show the patient how the risk changes as you run the blood pressure down, or change the cholesterol. It’s quite a powerful tool...’ - (Torley *et al.*, 2005)   - Knowledge about CVD risk, cardiovascular disease and its management. – (Collins *et al.*, 2017)   “[if] the cardiovascular risk is 20–30 [%], we can decrease it by normalization of blood pressure that’s high and the cholesterol level if it’s high, we can give him 3 months to 4 months diet and then recheck it. If it’s still high we can start statin to  reduce it.”– Doctor C - (Collins *et al.*, 2017)  ,   - Patients’ demand for risk assessment - (Van Steenkiste, Van Der Weijden, Stoffers, *et al.*, 2004; Torley *et al.*, 2005; Wan *et al.*, 2010)   ‘…But if I decide not to test them, it just means you spend 10 minutes explaining why it’s not necessary. Three months later they turn up at your office and say they want it anyway. That annoys me, especially since I’ve spent an extra 10 minutes on them. I’ve given up fighting it. (GP8)’ - (B van Steenkiste *et al.*, 2004)  If I want to know, I feel it’s my right to know – patient (Van Steenkiste, Van Der Weijden, Timmermans, *et al.*, 2004a)  In fact, I should believe what the doctor says, but then I start to think maybe it’s something else and then I ask for a little check-up – patient (Ben van Steenkiste *et al.*, 2004)   - Task allocation and shifting - (Doolan-Noble, Mann and Tracey, 2010)   “The doctors are more involved in risk assessment; the practice nurses are more concerned with the lifestyle management.” (F1)   - Patients’ relationship with the GP - (Wan *et al.*, 2010)   ‘If they’re just a ﬂoating patient coming to see you for a cough or cold or whatever it is, they probably have a regular doctor to go back to and follow through, but if you have some problems with your regular doctor, I’ll ﬁnd some time to go through it with you at least then you can get their conﬁdence and show them a reason...it depends how you approach them. (GP FG1)’ - (Wan *et al.*, 2010)  I think it’s being able to talk to your doctor, have faith in him and be comfortable with it. (Patient FG3) - (Wan *et al.*, 2010)   - Patients’ personal circumstances and experiences - (Doolan-Noble, Mann and Tracey, 2010)   “I’ve just felt that I was getting nowhere with, then there’s been something else that happened. For one it was another family member had a heart attack, and then it suddenly dawns on them, and all the work that you’ve done in the past is actually quite helpful.” (F2) - (Doolan-Noble, Mann and Tracey, 2010)   - Positive relationship with the practice and the presence of supportive programs - (Doolan-Noble, Mann and Tracey, 2010)   “There’s a really good pathway that was set up when, before we started this, which was, you know, the free GP visit… the dietician visit, the Green Prescription. And it’s really good.” (F2) - (Doolan-Noble, Mann and Tracey, 2010)   - Patients’ motivation/age – (Van Steenkiste, Van Der Weijden, Timmermans, *et al.*, 2004d)   The older you get, the less risk you want to run. I never used to think about that before, but now I do. Life’s too good to want to die now. Maybe, I should have a cholesterol check-up every 6 months now that I’m almost 60. It does not mean I’m old, but the risk of getting something is increasing. – patient - (Van Steenkiste, Van Der Weijden, Timmermans, *et al.*, 2004b)   - Presence of a support system (familial) for the patient - (Vaidya *et al.*, 2012)   It is a big step if you have got your family behind you. They have got to eat what I eat so there is no temptation. – patient (Vaidya *et al.*, 2012) | - Not knowing how to use the tool - (Oriol-Zerbe and Abholz, 2007; Sposito *et al.*, 2009a; Abaci, 2010; Dallongeville *et al.*, 2010; Roland E Schmieder, Goebel and Bramlage, 2012; Elisaf *et al.*, 2014; D. Byrne *et al.*, 2015; Collins *et al.*, 2017)   “Yes, they are 90 years old. When I look at them”,  “Okay, we will not do the risk assessment for this patient”. Because I don’t know how.”– Doctor D - (Collins *et al.*, 2017)  “I am not aware of any guide to manage risk factors.” [nurse] - (Collins *et al.*, 2017)   - Perception that prevention will not reduce total health care costs by clinicians - (Eichler *et al.*, 2007) - Perception that the tools would not reduce health care costs - (Tawfik *et al.*, 2015) - Defensive medicine - (Van Steenkiste, Van Der Weijden, Stoffers, *et al.*, 2004)   ‘If you see a cholesterol level of 8 in a 48-year-old man, your first reaction is a statin. But then, if you check the table, it may turn out he doesn’t need one. That is a different approach. But in the back of your mind you still think: what if he gets a heart attack and it then turns out he had a cholesterol level of 8 all the time and nothing was done about it? (GP6) - (Van Steenkiste, Van Der Weijden, Stoffers, *et al.*, 2004)  Suppose he gets a myocardial infarction in the future and then they find a cholesterol level of 8.0. There will always be someone who says: ‘‘You mean to say he never checked your cholesterol?’’ (GP15) - (Van Steenkiste, Van Der Weijden, Stoffers, *et al.*, 2004)   - The prediction rules are unclear - (Eichler *et al.*, 2007; S M Liew *et al.*, 2013)   GP18: ‘If someone’s on blood pressure treatment, do you say that treatment of blood pressure reduces [the assessed risk] by two thirds and therefore you need to modify for the third as not being attenuated? It’s all very, very grey, isn’t it?’ - (S M Liew *et al.*, 2013)   - Clinician perceptive of cardiovascular risk and disease -(Vaidya *et al.*, 2012; C Bonner *et al.*, 2013)   I’ve not used the cardiovascular risk assessment  terribly much  lately because we find that really at the end of the day the whole thing is just weight reduction. (ID36, male, 25 years’ experience) - (C Bonner *et al.*, 2013)  If someone’s got a known high cholesterol, I will treat that. If someone’s got a high blood pressure, I will treat that. I don’t need the risk calculator to tell me that I am meant to treat those risk factors. GP - (Vaidya *et al.*, 2012)   - Patients’ perceptions and understanding of cardiovascular risk and disease - (B van Steenkiste *et al.*, 2004; Wan *et al.*, 2008b; Doolan-Noble, Mann and Tracey, 2010; Carissa Bonner *et al.*, 2013; Collins *et al.*, 2017)   I do not think they can conclude from a blood sample whether you’ll get cardiovascular disease. I have seen it with my neighbour across the street. He had been in for a check-up and the hospital had said that everything was all right. Well, the day after that he had a heart attack and he was gone – patient (Van Steenkiste, Van Der Weijden, Stoffers, *et al.*, 2004)  I had 6.3 (mmol/l) serum cholesterol and my GP would not give me those pills. I know, there are all kinds ofother factors involved, but my uncle also had high cholesterol and no risk factors and he still had a heart attack. Give me those tablets anyway. - (Ben van Steenkiste *et al.*, 2004)  “Some of the Maori and Pacific Island… have this perception… that their parents died at such an age so, they’re not gonna make it past that age anyway, - (Doolan-Noble, Mann and Tracey, 2010)   - Tools are perceived as less superior to clinical judgement - (Torley *et al.*, 2005; Oriol-Zerbe and Abholz, 2007; A Imms, Quinn and Nelson, 2010; C Bonner *et al.*, 2013; Elustondo *et al.*, 2013)   [AR assessment] doesn’t take into account your family history, your weight, if you’re active or not . . . when you’ve been in this game for as many years as I have you like to get a big picture.  (ID22, male, 22 years’ experience) - (C Bonner *et al.*, 2013)  “I just go by my judgement more than by the risk assessment . . . rather than giving it in percentile I usually go by low risk, high risk” (clinical judgement) -(Carissa Bonner *et al.*, 2013)  ‘...You have to rely on your clinical gut feeling  about that patient. Taking all the information that you have gathered to date, put it all together and compute it in your mind and then decide how hard you are going to chase each of these risk factors...’ - (Torley *et al.*, 2005)   - The difficulty of communicating preventive care and self-management to patients by clinicians - (Doolan-Noble, Mann and Tracey, 2010)   “The concept of preventive care and ‘self-management’ is often a difficult one to impart.” (F3) - (Doolan-Noble, Mann and Tracey, 2010)  “I personally feel I could spend a lot of time on these patients (those at high risk), but the actual outcome would be possibly minimal.” (F3) - (Doolan-Noble, Mann and Tracey, 2010)   - Lack of motivation to use the tools - (Ferrante *et al.*, 2013)   “I am not motivated to read a guideline to treat my patients. I prefer to rely on my own knowledge and experience.” [attending clinician] - (Ferrante *et al.*, 2013)   - Health system’s priorities - (Doolan-Noble, Mann and Tracey, 2010; Ferrante *et al.*, 2013; Collins *et al.*, 2017)   “We are overwhelmed with urgent social and health problems and we can’t prioritize prevention activities.” [social worker] - (Ferrante *et al.*, 2013)  “Our model of care is based on satisfying the demand of patients, mainly acute problems, without a focus on chronic condition follow up.” [decision maker] - (Ferrante *et al.*, 2013)   - Manifestation of CVD risk - (Doolan-Noble, Mann and Tracey, 2010)   “…because they, don’t feel unwell. They don’t feel sick, so it’s really difficult to get their attention, motivation to get things. Like say they, they come with gout or something, you know they know…” (F3) - (Doolan-Noble, Mann and Tracey, 2010)   - Clinicians’ feeling of helplessness and unable to control patients behaviour with regard to prevention - (Eichler *et al.*, 2007)      - Clinicians’ ethical values - (Eichler *et al.*, 2007) - Patients’ fears and expectations of cardiovascular risk assessment - (Van Steenkiste, Van Der Weijden, Timmermans, *et al.*, 2004a; Wan *et al.*, 2010)   I do not need to know; that would only worry me more – patient - (Van Steenkiste, Van Der Weijden, Timmermans, *et al.*, 2004a)  Ignorance is bliss, as they say. The more you read about it, the more anxious you get. I know, I’m sticking my head in the sand. – patient - (Van Steenkiste, Van Der Weijden, Timmermans, *et al.*, 2004d)  I honestly believe I’ve been well looked after, but, ah, I don’t know, it’s hard to say. I hate going to the doctors to get me blood pressure tested. I really do. Just going there it goes up. (Patient FG2) - (Wan *et al.*, 2010) |
| **Process** – *Includes;*  Planning, engaging, executing, reflecting & evaluating | - Attracting and involving appropriate individuals - (Doolan-Noble, Mann and Tracey, 2010; Vaidya *et al.*, 2012)   It is a big step if you have got your family behind you. They have got to eat what I eat so there is no temptation. – patient - (Vaidya *et al.*, 2012) | - Health system’s priorities - (Doolan-Noble, Mann and Tracey, 2010; Ferrante *et al.*, 2013; Collins *et al.*, 2017)   “We are overwhelmed with urgent social and health problems and we can’t prioritize prevention activities.” [social worker] - (Ferrante *et al.*, 2013)  “Our model of care is based on satisfying the demand of patients, mainly acute problems, without a focus on chronic condition follow up.” [decision maker] - (Ferrante *et al.*, 2013)   - Physicians’ reflections on the use of tools - (Doolan-Noble, Mann and Tracey, 2010)   “I personally feel I could spend a lot of time on these patients (those at high risk), but the actual outcome would be possibly minimal.” (F3) - (Doolan-Noble, Mann and Tracey, 2010)   - Poor tool reviewing process - (Doolan-Noble, Mann and Tracey, 2010)   “I’ve seen three changes in long-term condition strategies, at a government level. And there is no consistency going down the line about how we should work within those strategies…” (F2) - (Doolan-Noble, Mann and Tracey, 2010)   - The perception that the tool maybe misused - (Eichler *et al.*, 2007; Ferrante *et al.*, 2013) - Lack of structures such as information systems to execute - (Ferrante *et al.*, 2013)   “We don’t have information systems in order to help us to follow up and manage patients with risk factors.” [attending family physician] - (Ferrante *et al.*, 2013)   - Unsustainable programs and funding to execute - (Doolan-Noble, Mann and Tracey, 2010)   Short-term contracts to run pilots, which just as you are gaining some traction the contract runs out, funding ceases and the programme falls over.” (F1) - (Doolan-Noble, Mann and Tracey, 2010) |
