# Supplementary material for: Factors influencing the implementation of cardiovascular risk scoring in primary care: a mixed-method systematic review
Source: Implement Sci. 2020 Jul 20;15:57. doi: 10.1186/s13012-020-01022-x (PMC7370418; doi:10.1186/s13012-020-01022-x)
Supplement: Supplementary file 6 — Additional File 6: Table S5-6. Quality Appraisal for Included studies [file 13012_2020_1022_MOESM6_ESM.docx]

**Table S6-7**. Quality Appraisal for Included studies

**S6. Quantitative studies**

| **Author** | **Abaci**  **2012** | **Bonnevie**  **2004** | **Byrne**  **2015** | **Dallongeville**  **2011** | **Eichler**  **2007** | **Elisaf**  **2014** | **Elustondo**  **2013** | **Imms**  **2010** | **Schmieder**  **2012** | **Shillinglaw**  **2012** | **Sposito**  **2009** | **Tawfik**  **2015** |
| --- | --- | --- | --- | --- | --- | --- | --- | --- | --- | --- | --- | --- |
| Were the aims/objectives of the study clear? | yes | yes | yes | yes | yes | yes | yes | yes | yes | yes | yes | yes |
| Was the study design appropriate for the stated aim? | yes | yes | yes | yes | yes | yes | yes | yes | yes | yes | yes | yes |
| Was the reference population clearly defined? Is it clear whom the research was about? | yes | yes | yes | yes | yes | yes | yes | yes | yes | yes | yes | yes |
| Was the sample frame taken from an appropriate population base so that it closely represented the reference population under investigation? | yes | yes | yes | yes | yes | yes | yes | yes | yes | yes | yes | yes |
| Was the selection process likely to select subjects/participants that were representative of the target/reference population under investigation? | yes | yes | yes | yes | yes | yes | yes | yes | yes | yes | yes | yes |
| Were measures undertaken to address and categorise non-responders? | no | no | no | no | no | no | no | yes | no | no | no | no |
| Were the risk factor and outcome variables measured appropriate to the aims of the study? | yes | yes | yes | yes | yes | yes | yes | yes | yes | yes | yes | yes |
| Were the risk factor and outcome variables measured correctly using instruments/measurements that had been trialled, piloted or published previously? | no | yes | yes | do not know | yes | yes | yes | yes | do not know | yes | do not know | yes |
| Is it clear what was used to determine statistical significance and/or precision estimates? (e.g. p-values, confidence intervals) | yes | yes | yes | yes | yes | yes | yes | yes | yes | yes | yes | yes |
| Were the methods (including the statistical methods) sufficiently described to enable them to be repeated? | yes | no | yes | yes | yes | yes | yes | yes | yes | yes | yes | yes |
| Were the basic data adequately described? | yes | yes | yes | yes | yes | yes | yes | yes | yes | yes | yes | yes |
| Does the response rate raise concerns about non-response bias?** | do not know | yes | yes | yes | yes | yes | yes | yes | yes | yes | do not know | no |
| If appropriate, was information about non-responders described? | no | no | no | no | no | no | no | no | no | no | no | no |
| Were the results internally consistent? | yes | no | yes | yes | yes | yes | no | yes | yes | yes | yes | yes |
| Were the limitations of the study discussed? | no | yes | yes | yes | yes | no | yes | yes | yes | yes | yes | no |
| Were there any funding sources or conflicts of interest that may affect the authors' interpretation of the results?** | no | no | no | yes | no | yes | do not know | no | yes | no | no | do not know |
| Was ethical approval or consent of participants attained? | yes | do not know | yes | yes | do not know | do not know | do not know | yes | yes | yes | do not know | do not know |

**S7. Qualitative studies**

| **Author** | **Bonner 2013** | **Doolan-Noble 2010** | **Liew 2013** | **Torley 2005** | **Vaidya 2012** | **Van Steenkiste 2004** | **Van Steenkiste 2004** | **Wan 2008** | **Wan 2010** |
| --- | --- | --- | --- | --- | --- | --- | --- | --- | --- |
| Is a qualitative approach appropriate? | yes | yes | yes | yes | yes | yes | yes | yes | yes |
| Is the study clear in what it seeks to do? | yes | yes | yes | yes | yes | yes | yes | yes | yes |
| How defensible/rigorous is the research design/methodology? | defensible | defensible | defensible | defensible | defensible | defensible | defensible | defensible | defensible |
| How well was the data collection carried out? | appropriately | appropriately | appropriately | appropriately | appropriately | appropriately | appropriately | appropriately | appropriately |
| Is the role of the researcher clearly described? | clearly described | clearly described | clearly described | unclear | clearly described | clearly described | clearly described | clearly described | clearly described |
| Is the context clearly described? | clearly described | clearly described | clearly described | clearly described | clearly described | clearly described | clearly described | clearly described | clearly described |
| Were the methods reliable? | yes | yes | yes | yes | yes | yes | yes | yes | yes |
| Is the data analysis sufficiently rigorous? | yes | yes | yes | yes | yes | yes | yes | yes | yes |
| Is the data 'rich'? | yes | yes | yes | yes | yes | yes | yes | yes | yes |
| Is the analysis reliable? | yes | yes | yes | yes | yes | yes | yes | yes | yes |
| Are the findings convincing? | yes | yes | yes | yes | yes | yes | yes | yes | yes |
| Are the findings relevant to the aims of the study? | yes | yes | yes | yes | yes | yes | yes | yes | yes |
| Conclusions? Is there adequate discussion of any limitations encountered? | yes | yes | yes | yes | yes | yes | yes | yes | yes |
| How clear and coherent is the reporting of ethics? | appropriate | appropriate | appropriate | inappropriate | appropriate | appropriate | appropriate | appropriate | appropriate |
| Is the study relevant? | yes | yes | yes | yes | yes | yes | yes | yes | yes |
| How well was the study conducted? ++, + or - | ++ | ++ | ++ | + | ++ | ++ | ++ | ++ | ++ |

**S8. Mixed methods studies**

| **Author** | **Collins 2017** | **Ferrante 2013** | **Kirby 2009** | **Oriol-Zerbe 2007** |
| --- | --- | --- | --- | --- |
| Are there clear qualitative and quantitative research questions (or objectives*), or a clear mixed methods question (or objective*)? | yes | yes | yes | yes |
| Do the collected data address the research question (objective)? | yes | yes | yes | yes |
| Are the sources of qualitative data (archives, documents, informants, observations) relevant to address the research question (objective)? | yes | yes | yes | yes |
| Is the process for analyzing qualitative data relevant to address the research question (objective)? | yes | yes | yes | yes |
| Is appropriate consideration given to how findings relate to the context, e.g., the setting, in which the data were collected? | yes | yes | yes | yes |
| Is appropriate consideration given to how findings relate to researchers’ influence, e.g., through their interactions with participants? | yes | yes | no | no |
| Are participants (organizations) recruited in a way that minimizes selection bias? | yes | yes | no | no |
| Is the sampling strategy relevant to address the quantitative research question (quantitative aspect of the mixed methods question)? | yes | yes | yes | yes |
| Is the sample representative of the population understudy? | yes | yes | yes | yes |
| Are measurements appropriate (clear origin, or validity known, or standard instrument)? | yes | yes | can’t tell | can’t tell |
| Is there an acceptable response rate (60% or above)? | n/a | n/a | yes | no |
| Is the mixed methods research design relevant to address the qualitative and quantitative research questions (or objectives), or the qualitative and quantitative aspects of the mixed methods question (or objective)? | yes | yes | yes | yes |
| Is the integration of qualitative and quantitative data (or results) relevant to address the research question(objective)? | yes | yes | yes | yes |
| Is appropriate consideration given to the limitations associated with this integration, e.g., the divergence of qualitative and quantitative data (or results*) in a triangulation design? | yes | yes | yes | yes |
| **Overall scoring metrics** | **100% (****)** | **100% (****)** | **75%(***)** | **75%(***)** |
