# Supplementary material for: Factors influencing the implementation of cardiovascular risk scoring in primary care: a mixed-method systematic review
Source: Implement Sci. 2020 Jul 20;15:57. doi: 10.1186/s13012-020-01022-x (PMC7370418; doi:10.1186/s13012-020-01022-x)
Supplement: Supplementary file 7 — Additional File 7: Table S7. Summary of the qualitative findings with their CERQual evidence profile and confidence assessments [file 13012_2020_1022_MOESM7_ESM.docx]

Table S8. Summary of the qualitative findings with their CERQual evidence profile and confidence assessments

| **Summary of the qualitative synthesis findings** | **Studies contributing to the review finding** | **Methodological limitations** | **Coherence** | **Adequacy** | **Relevance** | **CERQual assessment of confidence in the evidence** | **Explanation of CERQual assessment** |
| --- | --- | --- | --- | --- | --- | --- | --- |
| **Healthcare system and clinical setting:** In this review, this refers to the organisation of people, activities, resources, and priorities in primary care settings. The factors that emerged from this construct included resources, system and practice-level priorities, the practice culture of the clinical setting, and how the practice was organised. | | | | | | | |
| **Resources:**  This referred to both physical and human resources. An adequately resourced health system with a dedicated budget for the prevention of cardiovascular disease was viewed as a facilitator to the implementation of cardiovascular risk scoring. The absence of cardiovascular risk scoring tools in the consultation room was a barrier to implementation. | (Bonner et al. 2013, Doolan-Noble et al 2010) | No or very minor concerns about methodological limitations. | No or very minor concerns about coherence. | Serious concerns about adequacy – only two studies contributed to this and both had relatively thin data. However, this was judged to be a relatively simple and primarily descriptive finding.    Data was from only two high-income countries (Australia and New Zealand).    There was no data from low and middle-income countries. | No or very minor concerns about relevance – in one study, the specific risk scoring tools used was unclear. | Moderate confidence | The studies contributing to this review finding had no or very minor concerns about methodological limitations, coherence and relevance. There were serious concerns about adequacy, especially relating to the number of studies contributing to this finding and their geographical spread. |
| **System and practice-level priorities:**  In healthcare systems where prevention of cardiovascular disease was not seen as a priority for practice, cardiovascular risk scoring was implemented sub-optimally. Further, in some settings where prevention of cardiovascular disease was a priority, there was a conflict in deciding to whom this mandate belonged, i.e. clinicians versus local authorities. | (Doolan-Noble et al. 2010, Van Steenkiste, Van Der Weijden, Stoffers, et al 2004) | No or very minor concerns about methodological limitations. | No or very minor concerns about coherence. | Serious concerns about adequacy – only two studies contributed to this and both had relatively thin data. However, this was judged to be a relatively simple and primarily descriptive finding.    Data was from only two high-income countries (The Netherlands and New Zealand).    There was no data from low and middle-income countries. | No or very minor concerns about relevance. | Moderate confidence | The studies contributing to this review finding had no or very minor concerns about methodological limitations, coherence and relevance. There were serious concerns about adequacy, especially relating to the number of studies contributing to this finding and their geographical spread. |
| **Practice culture and organisation:**  This encompasses a set of routine activities that are characteristic of a healthcare system or clinical setting. Task shifting, re-allocation, and sharing in GP practices helped focus tasks into either risk assessment or risk management, necessitating the use of cardiovascular risk scoring tools by clinicians. Uptake of cardiovascular risk scoring was also seen in GP practices that had existing cardiovascular prevention care programs and pathways. However, adequate organisation of these prevention activities was of importance, i.e. staffed by appropriate persons and proximity to the clinical settings, i.e. for the clinician and the patient. Defensive medicine, a practice of recommending tests or treatment that is not necessarily the best option to the patient but as a function to protect the clinician against liability, was reported to be a barrier to cardiovascular risk scoring. Another barrier was the presence of disruptive professional hierarches, specialists or clinicians who ranked higher in practice were viewed in high regard even when they did not adhere to guidelines such as cardiovascular risk scoring. This set a precedent for practice in the clinical setting that junior clinicians did not feel empowered to question or disrupt. | (Doolan-Noble et al. 2010, Van Steenkiste, Van Der Weijden, Stoffers, et al 2004, Vaidya et al 2012) | No or very minor concerns about methodological limitations. | No or very minor concerns about coherence. | Serious concerns about adequacy – only three studies contributed to this and both had relatively thin data. However, this was judged to be a relatively simple and primarily descriptive finding.    Data was from only three high-income countries (The Netherlands, New Zealand and Australia).  There was no data from low and middle-income countries. | No or very minor concerns about relevance. | Moderate confidence | The studies contributing to this review finding had no or very minor concerns about methodological limitations, coherence and relevance. There were serious concerns about adequacy, especially relating to the number of studies contributing to this finding and their geographical spread. |
| **Users:** This encompassed factors that related to the direct and indirect users of cardiovascular risk scoring tools, i.e. clinicians, patients, and other stakeholders. These factors included the attributes of these users and the interactions between those involved. | | | | | | | |
| **Attributes of the users:**     1. **Both patients and clinicians**   For both patients and clinicians, the perception and understanding of cardiovascular risk, disease and its management was a significant driver for cardiovascular risk scoring. In most studies and for both clinicians and patients, there was a visible indication that cardiovascular risk, its assessment and management was marred with negative perceptions unsupported by evidence. For example, both clinicians and patients reported an understanding of cardiovascular risk focused on individual risk factors, i.e. relative risk as opposed to absolute or total risk. | (Bonner et al 2013, Doolan-Noble et al 2010, Van Steenkiste, Van Der Weijden, Stoffers, et al 2004, Torley et al 2005, Vaidya et al 2012, Wan et al 2008a) | No or very minor concerns about methodological limitations – one the study did not report ethical approval and unclearly described the role of the researcher. | No or very minor concerns about coherence. | Moderate concerns about adequacy - Data was from only three high-income countries (The Netherlands, New Zealand and Australia).    There was no data from low and middle-income countries. | No or very minor concerns about relevance - in one study the specific tools used was unclear | High confidence | The studies contributing to this review finding had no or very minor concerns about methodological limitations, coherence and relevance. There were moderate concerns about adequacy, especially relating to the geographical spread of the studies contributing to the finding. |
| 1. **Patients**   Personal circumstances and experiences of patients also influenced the uptake of cardiovascular risk scoring. These circumstances and experiences included the environment in which the patients lived, their socioeconomic standing, their fears, motivations and expectations of cardiovascular risk assessment and the support available for them. Patients’ priorities, which were set either by the patients themselves or the clinicians, leaned towards what was considered ‘urgent for the patient.’ In many cases, cardiovascular risk scoring was not viewed as urgent enough in this hierarchy. However, there were patients who demanded to be assessed for cardiovascular risk and this facilitated cardiovascular risk scoring.  Conversely, there were instances where patients demanded treatment regardless of their cardiovascular risk. | (Doolan-Noble et al. 2010, Van Steenkiste, Van Der Weijden, Timmermans, et al 2004, Torley et al 2005, Vaidya et al 2012, Wan et al 2010) | No or very minor concerns about methodological limitations – one the study did not report ethical approval and unclearly described the role of the researcher. | Moderate concerns about coherence – the extent of coherence unclear due to limited data. | Moderate concerns about adequacy - Data was from only three high-income countries (The Netherlands, New Zealand and Australia). There was no data from low and middle-income countries. | No or very minor concerns about relevance. | Moderate confidence | The studies contributing to this review finding had no or very minor concerns about methodological limitations and relevance. There were moderate concerns about adequacy and coherence, especially relating to the geographical spread of the studies and the limited data contributing to the finding. |
| 1. **Clinicians**   Clinicians expressed difficulty in communicating cardiovascular risk scores to their patients after risk assessment. This was coupled with challenges in offering prevention and self-management aids to patients. Knowledge about the tools and their benefits in clinical practice, such as improving care and bettering therapeutic decision making by clinicians facilitated the use of cardiovascular risk scoring. | (Doolan-Noble et al. 2010, Torley et al 2005, Vaidya et al 2012) | No or very minor concerns about methodological limitations – one the study did not report ethical approval and unclearly described the role of the researcher. | No or very minor concerns about coherence. | Serious concerns about adequacy – only three studies contributed to this and both had relatively thin data. However, this was judged to be a relatively simple and primarily descriptive finding.    Data was from only two high-income countries (Australia and New Zealand).    There was no data from low and middle-income countries. | No or very minor concerns about relevance | Moderate confidence | The studies contributing to this review finding had no or very minor concerns about methodological limitations, coherence and relevance. There were serious concerns about adequacy, especially relating to the number of studies contributing to this finding and their geographical spread. |
| 1. **Stakeholders**   Stakeholders, i.e. “decision-makers” who included policymakers and practice managers, were reported to lack interest or have conflicting interests regarding the prevention and management of cardiovascular risk. They lacked consensus on the use of risk scoring tools and had unsatisfactory processes in place to review continually risk-scoring tools used in practice. | (Doolan-Noble et al. 2010, Van Steenkiste, Van Der Weijden, Stoffers, et al 2004) | No or very minor concerns about methodological limitations. | No or very minor concerns about coherence. | Serious concerns about adequacy – only two studies contributed to this and both had relatively thin data. However, this was judged to be a relatively simple and primarily descriptive finding.    Data was from only two high-income countries (The Netherlands and New Zealand).    There was no data from low and middle-income countries. | No or very minor concerns about relevance. | Moderate confidence | The studies contributing to this review finding had no or very minor concerns about methodological limitations, coherence and relevance. There were serious concerns about adequacy, especially relating to the number of studies contributing to this finding and their geographical spread. |
| **Interactions between the users:**  This was reported as the relationships between clinicians, patients and other stakeholders. A supportive and longstanding relationship between the clinician and patient facilitated the use of cardiovascular risk scoring. This was because clinicians felt that for ‘floating’ patients, they already had a regular doctor to whom this responsibility of prevention belonged. Consequently, the clinicians in this case were only interested in dealing with the immediate complaint from the patient. The interactions between the clinicians and the other stakeholders were on a policy level where there was no consensus on the use of these tools. This was partially explained by the lack of communication and involvement in decision making of clinicians and other stakeholders. | (Doolan-Noble et al. 2010, Wan et al 2010) | No or very minor concerns about methodological limitations | No or very minor concerns about coherence | Serious concerns about adequacy – only two studies contributed to this and both had relatively thin data. However, this was judged to be a relatively simple and primarily descriptive finding.    Data was from only two high-income countries (Australia and New Zealand).    There was no data from low and middle income countries | No or very minor concerns about relevance | Moderate confidence | The studies contributing to this review finding had no or very minor concerns about methodological limitations, coherence and relevance. There were serious concerns about adequacy, especially relating to the number of studies contributing to this finding and their geographical spread. |
| **Cardiovascular risk scoring tools:** The factors influencing cardiovascular risk scoring were linked to the characteristics of the tools, the perceived role of the tools in clinical practice and the evidence of efficiency and effectiveness. | | | | | | | |
| **Characteristics of these tools**:  The use of charts, calculators and colour codes was reported to facilitate cardiovascular risk scoring due to the perceived ease of use. Besides, having these tools incorporated into existing clinical systems as software or web-based applications was facilitative. This was while some studies reported that these tools were complex to use, to explain to patients and that they had technical problems. For example, some of the tools did not communicate with other clinical programs. Other studies pointed out that the tools outdated rapidly and consequently did not include all the important risk factors needed to assess for cardiovascular risk. | (Doolan-Noble et al. 2010, Liew et al. 2013, Van Steenkiste, Van Der Weijden, Stoffers, et al 2004, Vaidya et al 2012) | No or very minor concerns about methodological limitations | No or very minor concerns about coherence | Moderate concerns about adequacy – only four studies contributed to this and all had relatively thin data. However, this was judged to be a relatively simple and primarily descriptive finding.    Data was from only four high-income countries (Australia, New Zealand, United Kingdom and The Netherlands).    There was no data from low and middle income countries | No or very minor concerns about relevance | High confidence | The studies contributing to this review finding had no or very minor concerns about methodological limitations, coherence and relevance. There was moderate concerns about adequacy, especially relating to the number of studies contributing to this finding and the lack of data from low and middle-income countries. |
| **Their perceived role:**  The perceived role of these tools in clinical practice was both a facilitator and a barrier to their use. For example, clinicians who used the tools saw it as helpful in understanding risk, motivating patients, improving follow up, educating patients, and as a checklist for risk assessment. Others saw it as an interference to the clinician’s decision-making process. This, perhaps, is in relation to clinicians considering these tools to be less superior to their clinical judgement. | (Bonner et al 2013, Torley et al 2005, Wan et al. 2008a) | No or very minor concerns about methodological limitations – one the study did not report ethical approval and unclearly described the role of the researcher | No or very minor concerns about coherence | Serious concerns about adequacy – only three studies contributed to this and both had relatively thin data. However, this was judged to be a relatively simple and primarily descriptive finding.    Data was from only one high-income country (Australia).    There was no data from low and middle income countries | No or very minor concerns about relevance | Moderate confidence | The studies contributing to this review finding had no or very minor concerns about methodological limitations, coherence and relevance. There were serious concerns about adequacy, especially relating to the number of studies contributing to this finding and their geographical spread. |
| **Evidence of clinical and cost effectiveness:**  Unclear prediction rules were a barrier to using cardiovascular risk scoring tools, as this was associated with prediction inaccuracies. However, providing evidence that these tools were accurate in predicting risk, that they included the main risk factors for cardiovascular disease and that they led to better therapeutic decisions was facilitative. | (Bonner et al 2013, Liew et al 2013, Wan et al. 2008b) | No or very minor concerns about methodological limitations | No or very minor concerns about coherence | Serious concerns about adequacy – only three studies contributed to this and they had relatively thin data. However, this was judged to be a relatively simple and primarily descriptive finding.    Data was from only two high-income countries (The United Kingdom and Australia).    There was no data from low and middle income countries | No or very minor concerns about relevance | Moderate confidence | The studies contributing to this review finding had no or very minor concerns about methodological limitations, coherence and relevance. There were serious concerns about adequacy especially relating to the number of studies contributing to this finding and their geographical |
